# The Acute and Chronic Effects of Implementing Velocity Loss Thresholds During Resistance Training: A Systematic Review, Meta-Analysis, and Critical Evaluation of the Literature

**DOI:** 10.1007/s40279-022-01754-4

**Published:** 2022-09-30

**Authors:** Ivan Jukic, Alejandro Pérez Castilla, Amador García Ramos, Bas Van Hooren, Michael R. McGuigan, Eric R. Helms

**Affiliations:** 1 Sport Performance Research Institute New Zealand (SPRINZ), Auckland University of Technology, Auckland, New Zealand; 2 School of Engineering, Computer and Mathematical Sciences, Auckland University of Technology, Auckland, New Zealand; 3 Department of Physical Education and Sport, Faculty of Sport Sciences, University of Granada, Granada, Spain; 4 Department of Sports Sciences and Physical Conditioning, Faculty of Education, Universidad Católica de la Santísima Concepción, Concepción, Chile; 5 Department of Nutrition and Movement Sciences, NUTRIM School of Nutrition and Translational Research in Metabolism, Maastricht University Medical Centre+, Maastricht, The Netherlands

## Abstract

**Background:**

Velocity loss (VL) experienced in a set during resistance training is often monitored to control training volume and quantify acute fatigue responses. Accordingly, various VL thresholds are used to prescribe resistance training and target different training adaptations. However, there are inconsistencies in the current body of evidence regarding the magnitude of the acute and chronic responses to the amount of VL experienced during resistance training.

**Objective:**

The aim of this systematic review was to (1) evaluate the acute training volume, neuromuscular, metabolic, and perceptual responses to the amount of VL experienced during resistance training; (2) synthesize the available evidence on the chronic effects of different VL thresholds on training adaptations; and (3) provide an overview of the factors that might differentially influence the magnitude of specific acute and chronic responses to VL during resistance training.

**Methods:**

This review was performed using the Preferred Reporting Items for Systematic Reviews and Meta-Analyses (PRISMA) guidelines. Five databases were searched, and studies were included if they were written in English, prescribed resistance training using VL, and evaluated at least one (1) acute training volume, neuromuscular, metabolic, or perceptual response or (2) training adaptation. Risk of bias was assessed using a modified Cochrane Collaboration’s tool for assessing the risk of bias in randomized trials. Multilevel and multivariate meta-regressions were performed where possible.

**Results:**

Eighteen acute and 19 longitudinal studies met the inclusion criteria, of which only one had more than one risk of bias item assessed as high risk. Based on the included acute studies, it seems that the number of repetitions per set, blood lactate concentration, and rating of perceived exertion generally increase, while countermovement jump height, running sprint times, and velocity against fixed loads generally decrease as VL increases. However, the magnitude of these effects seems to be influenced, among other factors, by the exercise and load used. Regarding training adaptations, VL experienced during resistance training did not influence muscle strength and endurance gains. Increases in VL were associated with increases in hypertrophy (*b* = 0.006; 95% confidence interval [CI] 0.001, 0.012), but negatively affected countermovement jump (*b* = − 0.040; 95% CI − 0.079, − 0.001), sprint (*b* = 0.001; 95% CI 0.001, 0.002), and velocity against submaximal load performance (*b* = − 0.018; 95% CI − 0.029, − 0.006).

**Conclusions:**

A graded relationship exists between VL experienced during a set and acute training volume, neuromuscular, metabolic, and perceptual responses to resistance training. However, choice of exercise, load, and individual trainee characteristics (e.g., training history) seem to modulate these relationships. The choice of VL threshold does not seem to affect strength and muscle endurance gains whereas higher VL thresholds are superior for enhancing hypertrophy, and lower VL thresholds are superior for jumping, sprinting, and velocity against submaximal loads performance.

**Clinical Trial Registration:**

The original protocol was prospectively registered (https://osf.io/q4acs/) with the Open Science Framework.

**Supplementary Information:**

The online version contains supplementary material available at 10.1007/s40279-022-01754-4.

## Key Points

**Table:** 

A graded relationship exists between velocity loss (VL) experienced during a set and acute training volume, neuromuscular, metabolic, and perceptual responses to resistance training with factors such as type of exercise, loads used, and individual characteristics of a trainee seeming to modulate these relationships.
Factors that can specifically affect the consistency of VL determination include reference repetitions, velocity variables (e.g., mean or peak), and criteria for set termination after VL has been exceeded, all of which should be considered when implementing VL in practice.
The amount of VL experienced during resistance training does not seem to affect strength and muscle endurance gains whereas higher VL may be superior when the aim is to induce hypertrophy. Allowing only low to moderate VL during resistance training seems to be a viable strategy for optimizing jumping, sprinting, and velocity against submaximal loads performance.
As higher VL experienced during resistance training could interfere with the ability to rapidly produce force, cause a reduction in the expression of fast-twitch muscle fibers, and prolong recovery from resistance training, low to moderate VL could be recommended to optimize strength and power training adaptations as well as the performance of sport-specific tasks. However, if hypertrophy is also the goal, more of the prescribed sets could utilize moderate VLs, or more total sets with low to moderate VL could be performed.

## Introduction

Resistance training (RT) can produce many adaptations including strength, power, hypertrophy, and endurance, and for this reason plays an integral role in many long-term athlete development programs. While these adaptations may improve performance of athletic tasks such as jumping, sprinting, and change of direction [1, 2], resistance training also plays an important role in injury prevention and rehabilitation and has numerous beneficial effects on health and quality of life [3–6]. Designing an effective RT program requires careful consideration of many training variables such as the choice and order of the exercises, load, repetition range, volume, rest, intended velocity, and set structure configuration. Among these, training load and volume appear to be the most important training variables dictating the type and extent of acute and chronic adaptations to RT [7–9]. Traditionally, load is prescribed relative to a one-repetition maximum (%1RM) while RT volume is manipulated by modifying the total number of sets performed and/or the number of repetitions performed per set. Although this approach is relatively simple and efficient, it does not account for physiological and psychological stressors that might affect an individual’s day-to-day RT performance as well as inter-individual variability in RT performance [10]. For instance, load prescription based on %1RM might be less accurate as maximal strength can fluctuate daily [11] when an individual is fatigued or significantly increase within a few weeks because of training adaptations [12]. Further, the number of repetitions that can be completed with a given %1RM is highly variable as it is both individual and exercise specific [13, 14]. In this regard, sport scientists have explored velocity-based training approaches to load and volume prescription as an alternative method that may circumvent some of these limitations [10].

Load and volume prescription with velocity-based training rests on the premise that there is an inverse linear relationship between barbell velocity and %1RM; heavier loads cannot be lifted with the same velocity as lighter loads [10]. Furthermore, if an exercise is performed with maximal concentric effort and fatigue ensues, barbell velocity inevitably decreases [14]. Indeed, very strong correlations exist between intra-set velocity loss (VL) and mechanical, perceptual, and metabolic markers of fatigue [14–16], as well as between VL and the number of completed repetitions relative to the maximum number of repetitions possible in a set [15, 17]. For instance, in the squat, terminating a set after reaching 20% VL would typically result in 50% of the possible repetitions being completed [14], whereas a 40 or 50% VL would result in repetitions performed to, or very near, muscle failure [18]. Therefore, VL may be used as an indicator of fatigue during RT, and thus, may be used to regulate volume and proximity to failure with reasonable precision [14–17, 19].

Indeed, several studies have been conducted to investigate the acute effects of different VL thresholds on various correlates and markers of fatigue and generally reported nearly linear increases in fatigue as VL increased across the sets [14–16, 20]. For instance, Rodríguez-Rosell et al. [16] observed a gradual increase in blood lactate accumulation as VL thresholds increased from 10 to 45% and from 15 to 55% during sets of back squat and bench press, respectively. Weakley et al. [21] observed the same trend with 10, 20, and 30% VL, while also reporting a gradual decline in countermovement jump height and gradual increases in perceived exertion of the lower limbs and breathlessness after each set. Finally, Pareja-Blanco et al. [22] reported that for a given %1RM, a higher magnitude of VL in a set results in greater impairment of neuromuscular performance immediately after the training session and slower post-exercise recovery 24 and 48 h later. While these findings illustrate the utility of monitoring VL for RT prescription, some researchers suggested that the effects of different VL experienced during a set on the magnitude of neuromuscular, metabolic, and perceptual fatigue accumulation might depend upon the exercise and load used [16, 23]. In addition, the magnitude of VL itself could be affected by the reference repetition for determining VL (i.e., first vs fastest) [24] and the criteria for set termination (e.g., terminating a set after one or more repetitions passed below a certain VL threshold) [24]. Finally, although VL is frequently used to prescribe RT volume, the exact number of repetitions performed before reaching certain VL thresholds is also likely affected by the load and exercise used, as well as inter-individual variability and perhaps the reliability of velocity monitoring devices. Despite these limitations, different VL thresholds are often used with the aim of creating more homogeneous RT stimuli among individuals, which in turn are thought to lead to more consistent and enhanced long-term adaptations [10], although more research is needed to confirm these speculations.

Considerable evidence is accumulating from longitudinal studies (> 4 weeks in duration) comparing the effectiveness of different VL thresholds to one another on muscular strength, hypertrophy, and endurance as well as the performance of athletic tasks. In this regard, it has been suggested that the selected VL threshold can modulate adaptations to training in a dose–response manner [18, 25–27]. For instance, Pareja-Blanco et al. [26] recently showed that there might be an upper and lower VL threshold that should be prescribed during RT to induce optimal training adaptations, indicating that the dose–response relationship might follow an inverted U shape. Thus, it was concluded that low to moderate VL thresholds (i.e., 10 and 20%) should be chosen to optimize adaptations to RT because VL thresholds lower than 10% induced levels of fatigue that were too low to maximize adaptations, whereas high VL thresholds (i.e., > 40%) did not promote further strength or hypertrophy, and negatively affected the improvement of athletic tasks compared with moderate VL thresholds [26]. However, not all studies support this as similar improvements in maximal strength [28, 29], hypertrophy [29], and sprinting and jumping performance [28] were observed between lower and higher VL thresholds. To further confound matters, other factors such as training duration, choice of exercise, load, and participant strength levels likely moderate the effects of VL thresholds on various training adaptations.

In light of these considerations and inconsistencies in the scientific literature, there is a clear need for a comprehensive review and synthesis of the available evidence. Therefore, the aim of this systematic review and meta-analysis was to synthesize the available evidence on (1) the acute effects of different VL thresholds on markers of fatigue and number of repetitions per set during RT and (2) the chronic effects of different VL thresholds on training adaptations. This review also aimed to provide an overview of the factors that might differentially influence the magnitude of acute and chronic responses to different VL thresholds, thus providing a more nuanced assessment of the dose–response relationship between VL, acute fatigue accumulation, and various training adaptations. Such information is important to inform RT prescription strategies based on VL thresholds, ultimately allowing for better fatigue management and attainment of intended training adaptations.

## Methods

### Registration of Systematic Review Protocol

A systematic review of the literature was performed according to the guidelines in the *Cochrane Handbook for Systematic Reviews of Interventions* (version 6.0) and following the 2020 checklist for the Preferred Reporting Items for Systematic Reviews and Meta-Analyses (PRISMA) [30]. The original protocol was prospectively registered at the Open Science Framework (https://osf.io/q4acs/). The protocol registration occurred after searches were conducted, but before screening was completed and data extraction started.

### Eligibility Criteria

All studies included met the following inclusion criteria: (1) the study was published in English; (2) evaluated the acute effects of one or more VL thresholds during RT on neuromuscular, metabolic and perceptual markers of fatigue, and/or examined their chronic effects on muscular strength, hypertrophy, endurance or power adaptations; (3) RT was prescribed using VL thresholds; (4) intensity of load (%1RM) and frequency were matched between conditions; (5) participants had no known medical condition or injury; (6) in acute studies, neuromuscular, metabolic, or perceptual responses (and variability thereof) to these thresholds were considered; (7) in longitudinal studies, the outcomes were assessed pre-intervention and post-intervention for muscular strength with a repetition maximum component, or maximum voluntary contraction test, hypertrophy (lean body mass changes or changes at the muscle level), endurance (total repetitions performed or mechanical work), and power adaptations (jump height, sprint and change of direction times, or velocity at a fixed load); and (8) training interventions in longitudinal studies lasted a minimum of 4 weeks.

### Information Sources and Search Strategy

A PICO strategy consisting of terms for different VL thresholds, RT, and neuromuscular, perceptual, and metabolic outcomes as well as muscular strength, endurance, hypertrophy, and power adaptations was used to build search criteria for electronic databases. To ensure the inclusiveness of the search terms, the Word Frequency Analyser tool (http://sr-accelerator.com/#/help/wordfreq) was used to suggest potentially relevant search terms [31]. In addition, the Research refiner tool (https://ielab-sysrev2.uqcloud.net/) was used to optimize the sensitivity and specificity of the search for PubMed, while the Polyglot Search Translator Tool (https://sr-accelerator.com/#/polyglot) was used to adapt the search to other databases [31, 32]. The search string used for MEDLINE/PubMed is reported in the Electronic Supplementary Material (ESM). The following bibliographic databases were searched from inception to 6 December, 2020: PubMed/MEDLINE, SCOPUS, CINAHL (Cumulative Index to Nursing and Allied Health), SPORTDiscus, and Web of Science. No year restrictions were applied. Secondary searches included: (a) screening the reference lists of all included studies and relevant review papers; (b) examining the studies that cited the included studies (i.e., forward citation tracking) through Google Scholar; and (c) search alerts to monitor any new search results after the date of the last search up to 21 June, 2022.

### Study Selection

Duplicate references were first removed using the EndNote reference manager (version X9.0.3; Clarivate Analytics, Philadelphia, PA, USA). Two authors (IJ and AGR) then independently screened titles and abstracts to determine initial eligibility using the systematic review software Rayyan. Authors were blinded to avoid bias during this process. Thereafter, the authors (IJ and AGR) independently screened the full texts to determine inclusion eligibility. Disagreements over eligibility at any stage were resolved through discussion, or with a third reviewer (BVH) when required.

### Data Extraction

The following data were extracted from the included studies into an Excel spreadsheet: (1) study design and identification information; (2) adherence and study duration; (3) sample size; (4) participants’ age, body mass, height, sex, strength levels, and training experience; (5) relevant information regarding VL thresholds used, including various methodological factors (e.g., reference repetition, velocity variable, prescription method); and (6) means and standard deviations as well as raw mean changes and standard deviations of changes for pre-intervention and post-intervention assessments of the relevant outcome measures. If insufficient data were reported, the authors of those studies were contacted by e-mail. Web Plot Digitizer software (Version 4.1; https://automeris.io/WebPlotDigitizer/) was used to extract data from figures when the authors did not report or provide the data. Data extraction was completed independently by three authors (IJ, AGR, and APC) using two pilot-tested forms (one for acute and one for longitudinal studies) on five randomly selected studies that were then modified accordingly. Coding files were cross-checked between the authors, and any differences were resolved via discussion and agreement, or with a fourth reviewer (BVH).

### Risk of Bias Assessment

Risk of bias assessment was performed using a modified Cochrane Collaboration tool for assessing the risk of bias in randomized trials [33]. Modifications included removal of the performance bias and blinding of outcome assessment bias criteria and adding effort bias, feedback bias, training prescription bias (for longitudinal studies only), outcome assessment bias, and familiarization bias. Blinding of outcome assessment bias was excluded as visual and verbal velocity feedback were used in the reviewed studies to ensure participants’ maximal intent, which improves the reliability of performance. Similar to previous systematic reviews and meta-analyses on exercise intervention studies [34, 35], the performance bias criterion was removed because it is impossible to blind participants and personnel in supervised exercise intervention studies. Assessments were completed independently by two reviewers (IJ and ERH) while any observed differences were resolved via discussion and agreement before merging the scores into a single spreadsheet.

### Statistical Analysis

#### Acute Effects of Velocity Loss Thresholds

While we a priori planned to examine the acute effects of different VL thresholds during RT on repetition volume, neuromuscular, metabolic, and perceptual responses, and potential moderating effects of exercise, training prescription method, reference repetition for VL calculation, load, and strength levels of individuals, this was not done because of one or more of the following reasons: (1) a low number of studies reporting these outcomes; (2) a large amount of missing data; and (3) authors’ non-responsiveness to data request e-mails or refusal to provide data necessary for calculating effect sizes (usually baseline means and standard deviations, standard deviations of difference scores, or pre-post correlations). Attempts were made to circumvent these issues while making assumptions about baseline data based on other studies and estimating missing data using the data that were available following the procedures outlined by Elbourne et al. [36] and Borenstein et al. [37]. However, these procedures often resulted in spurious calculations (e.g., *r* > 1) that discouraged us from pursuing the meta-analysis. Nevertheless, to aid the interpretation of the findings, we used the data reported in the original studies and created visualisations that could be used to observe potential trends and interactions between the variables. Importantly, this was done only when a whole range of VL thresholds were investigated for a given outcome.

#### Chronic Effects of Velocity Loss Thresholds

The nature of our research question with regard to chronic effects of different VL thresholds on muscle strength, hypertrophy, and endurance, as well as sprint, countermovement jump, and velocity against submaximal load performance required the inclusion of a VL threshold, as a continuous moderator, in all meta-analytic models. This was needed as each study compared different VL thresholds to one another, rather than to no training at all (i.e., no control groups were included in the studies).

##### Calculation of Effect Size and Variance

Standardized mean changes were computed to quantify the effect of the intervention using different VL thresholds relative to the baseline, thereby permitting synthesis of the same outcome variable (e.g., strength, hypertrophy) from different procedures or scales. However, raw mean changes were computed and used as a summary measure of effect size when a given outcome was assessed using the same procedure or scale to aid the interpretation of the findings. Standardized mean changes for each group was calculated as the difference between post-test and pre-test scores, divided by the pre-test standard deviation with an adjustment (*C*) for a small sample bias [38–40]:

$${\text{SMC}} = C\left( {\frac{{M_{{{\text{post}}}} {-} M_{{{\text{pre}}}} }}{{{\text{SD}}_{{{\text{pre}}}} }}} \right); \quad C_{j} = 1 - \frac{3}{{4\left( {n_{j} - 1} \right) - 1}}.$$

The standardized mean change magnitude was interpreted as: small (0.20–0.49), moderate (0.50–0.79), and large (> 0.80) [41].

No studies reported the pre-intervention to post-intervention correlations required to determine the variance. Therefore, when the authors did not provide correlations upon our request, standard deviations of the pre-intervention to post-intervention change were used to calculate pre-to-post correlations using the following formula:

$${r}_{j}= \frac{{\mathrm{SD}}_{j,\mathrm{pre}}^{2} + {\mathrm{SD}}_{j,\mathrm{post} }^{2}- {\mathrm{SD}}_{j,\mathrm{ change}}^{2}}{2 \times {\mathrm{SD}}_{j,\mathrm{ pre}} \times {\mathrm{SD}}_{j,\mathrm{post}}}.$$

The corresponding authors were contacted when the standard deviations of the pre-intervention to post-intervention change were not reported. Of all the corresponding authors, one did not respond [42], whereas the corresponding author of the following studies included in this review [43–45] declined to provide the requested data. The other authors provided the necessary data to calculate the variance. For the missing standard deviation of the pre-intervention to post-intervention change, the median correlation using all other studies for a given outcome was imputed. This ensured that the maximum number of studies were included. The variability in designs among eligible studies required several decisions to ensure the data could be appropriately combined for the calculation of effect sizes. These decisions are detailed in the ESM.

##### Statistical Synthesis of Effect Sizes

Most studies in the quantitative part of the synthesis (81.2%) provided two or more effect sizes while comparing the effects of different VL thresholds. Effect sizes from the same study are likely more similar than effect sizes from different studies [46]. Thus, the inclusion of multiple effect sizes from a single study violates the assumption of independence in effect sizes in traditional meta-analyses (e.g., [47, 48]). As such, a three-level meta-analysis (i.e., a multilevel model) was used to account for dependencies among effect sizes from the same study [49]. A multilevel meta-analysis accounts for the hierarchical nature of the data (e.g., effect sizes nested within studies) and, in so doing, the extraction of multiple effects from each study preserves information improving statistical power [46]. This approach also decomposes the variance components of the pooled effect into sampling variance of the observed effect sizes (level 1), and variance within (level 2) and between studies (level 3) [47]. A multilevel meta-analysis was conducted for every outcome separately except for velocity at submaximal loads. For velocity against submaximal (low and moderate) load outcomes, a multivariate mixed-effects meta-regression was performed. In addition, cluster-robust variance estimation methods [50] with small-sample adjustments [51] were implemented to calculate standard errors of the overall effect size estimates, with clustering at the study level. This was done because (1) most studies reported changes in velocity against low and moderate loads and (2) all these studies reported multiple effect sizes for both sub-outcomes (i.e., moderate and low loads), and different VL thresholds. Therefore, these two sub-outcomes were highly correlated as the data from the same participants were analyzed multiple times for both sub-outcomes, giving rise to both hierarchical and correlated effects for this outcome. The correlation (*ρ*) between moderate and low loads was assumed to be 0.6. Observations were weighted by the inverse of the sampling variance, and all (final) model parameters were estimated by the restricted maximum likelihood estimation method. Tests of individual coefficients in all models, and their corresponding confidence intervals, were based on a t-distribution. Multilevel and multivariate models were fitted in R language and environment for statistical computing (version 4.0.5; R Core Team, Vienna, Austria) using the *metafor* package [52], while the cluster-robust variance estimation method was implemented using the *clubSandwich* package [53].

##### Moderator and Sensitivity Analyses

All meta-analytic models (i.e., multilevel and multivariate mixed-effects meta-regressions) included VL as a continuous moderator. Further, other theoretically relevant moderators were included when (1) the number of effect sizes was sufficient (at least eight to ten per moderator) and (2) the range of observations (or levels in case of categorical predictors) was not very narrow or identical among the studies. These moderators included study duration (continuous predictor), exercise (upper or lower body exercise), loads (higher and lower than 70% of 1RM), and strength levels (continuous predictor). The exercise moderator was categorized because back squat and bench press were the most prevalent exercises among the studies. In addition, the loads moderator was categorized as the majority of primary studies used progressive overloads across the weeks and averaging these loads to a single number might not accurately represent the loads used in a given study. Because of the inclusion of both fixed and random effects, restricted maximum likelihood estimation was used to evaluate the final models for each outcome. Furthermore, their contribution—and the contribution of modeled interactions among predictors—to the explanatory power of any of the explored models was examined using a likelihood ratio test, deviance statistic, and Akaike information criterion score for small sample sizes before selecting the final model to obtain the best fit while maintaining model parsimony. During this process, models were fitted—and subsequently compared—using the maximum likelihood method as likelihood ratio tests cannot be used to compare models with nested fixed effects using restricted maximum likelihood estimation estimates [54]. Finally, a dose–response relationship considering (1) individual study effect sizes; (2) average effect sizes of individual VL thresholds; and (3) average effect sizes of low (15% VL), moderate (> 15% < 30% VL), and high (> 30% VL) grouped VL thresholds was also evaluated for each outcome to aid interpretation of the findings.

For all meta-analytic models, *Leverage*, *outlier*, and *influential* case diagnostics were performed by calculating *hat*, *Cook’s distance,* and *studentized residuals*, respectively [55–57]. Cases were red flagged with their hat and Cook’s distance’s values greater than three times their respective mean, and with a studentized residual’s value greater than 3, in absolute values. For the multivariate model investigating the effects of VL thresholds on velocity against submaximal loads, a range of correlations between the outcomes were imputed (*ρ* = 0.4–0.8) to ensure the robustness of the estimates.

Publication bias was not assessed as we were not interested in the effects of training interventions in individual studies, but rather as a moderator effect of VL thresholds examined within those studies. In addition, there was no reason to expect that a certain training intervention would not result in a significant improvement over time in at least some of the outcomes given the absence of control groups (interpreted here as groups who would not train at all).

##### Statistical Heterogeneity

As all multilevel models included moderators (i.e., VL), statistical indices of heterogeneity were evaluated using *I*^2^ and *τ*^2^, which represented relative and absolute values of residual heterogeneity or the amount of the unaccounted for variability that is due to residual heterogeneity [58]. This heterogeneity was then partitioned across two levels (i.e., within-study and between-study heterogeneity). Importantly, for all multilevel models, the estimated proportional reduction in the total variance was computed using the variance accounted for, a pseudo *R*^2^ value (i.e., the amount of heterogeneity accounted for by the moderators) [59]. For the cluster-robust multivariate meta-regression, the amount of heterogeneity (*τ*^2^) for each outcome was calculated as well as the correlation between the outcomes (*ρ*).

## Results

### Search Results

**Fig. 1:**
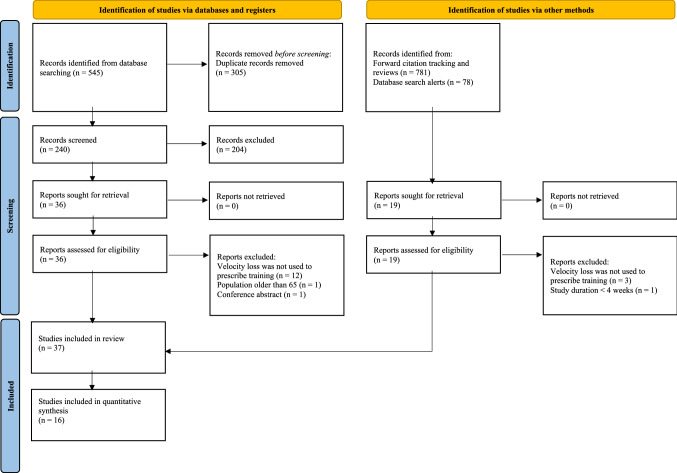
Literature search flow chart. *n* number of studies

The primary search yielded 545 results, of which 22 met the inclusion criteria. Forward citation tracking as well as monitoring the newly published relevant literature yielded an additional 15 studies, resulting in 37 studies included in this review. The stages of the search and study selection process are presented in Fig. 1.

### Study Characteristics

Out of 37 studies included, 18 were randomized cross-over acute studies, and 19 were training intervention studies. The total number of participants pooled across studies was 846 (767 were male and 69 were female). However, upon inspection, it was clear data from the same participants were used in multiple studies [20, 60–62]. This reduced the total number of participants to 735 (656 were male and 69 were female). Only five studies [29, 63–66] included male and female participants, two of them only female [67, 68] while the rest included only male participants. Back squat was the most frequently used exercise (26 studies), followed by bench press (12 studies), deadlift (two studies), bench pull, overhead press, leg press, loaded countermovement jump, and pull-up (one study each). Eleven studies used free-weight exercises, while the remaining used a Smith machine. A large range of VL thresholds were examined (0–55%) with 10, 20, 30, and 40% VL thresholds being the most frequent (ten or more studies each). In addition, participants with a large range of strength levels (1RM/body mass) were examined with the average lower and upper body maximal strength of participants being 1.48 (range 0.7–2.2) and 1.15 (range 0.65–1.56) times body mass, respectively. Velocity loss thresholds were prescribed using the first repetition (14 studies), and the fastest repetition (23 studies) of the set as the reference point. Load was prescribed with percentage of 1RM (12 studies), generalized load-velocity profiles (22 studies), and individualized load-velocity profiles (four studies). For longitudinal studies, the median study duration was 8 weeks (range 4–12). A more comprehensive description of the participants and the included studies can be found in Tables 1, 2, and 3.

**Table 1:** Study characteristics

Study	Study design	Participants	Sex: M/F	Age (years)	Height (cm)	Mass (kg)	Training experience (subjective description; years of RT experience; relative strength levels BM/1RM; exercise)
Alcazar et al. (2021) [60]	Chronic: randomly assigned	VL0: *n* = 14 VL10: *n* = 14 VL20: *n* = 13 VL40: *n* = 16	58/0	24 ± 4	175 ± 6	76 ± 10	Resistance trained individuals; 1.5–4; 1.3 ± 0.2; Smith machine full back-squat
Andersen et al. (2021) [29]	Chronic: randomly assigned	VL15: *n* = 10 VL30: *n* = 10	3/7	23 ± 4	171 ± 8	68 ± 9	Healthy individuals; 4.5 ± 0.7; 1.2 ± 0.2; leg press
Banyard et al. (2019) [77]	Acute: randomized crossover design	VL20_FS_: *n* = 15 VL20_VS_: *n* = 15	15/0	25 ± 4	180 ± 7	84 ± 11	Resistance trained individuals; 7 ± 2; 1.8 ± 0.3; free-weight full back-squat
Dorrel et al. (2020) [75]	Chronic: randomly assigned	VL20: *n* = 8	38/0	23 ± 5	180 ± 6	89 ± 13	Resistance-trained individuals; ≥ 2; 1.5 ± 0.3, 1.1 ± 0.2, 0.7 ± 0.1, and 2.0 ± 0.3, free-weight perceived optimum depth back-squat, free-weight bench press, free-weight strict overhead press, and free-weight deadlift, respectively
Fernandez-Ortega et al. (2020) [67]	Chronic: randomly assigned	VG: *n* = 15	0/15	14 ± 1	157 ± 7	47 ± 5	Adolescent soccer players; unexperienced; 0.7 ± 0.1; Smith machine full back-squat
Galiano et al. (2020) [78]	Chronic: randomly assigned	VL5: *n* = 15 VL20: *n* = 13	28/0	22 ± 3 24 ± 3	175 ± 5 177 ± 5	73 ± 11 76 ± 9	Physically active individuals; ≥ 1.5; 1.3 ± 0.2; Smith machine full back-squat
García-Sillero et al. (2021) [69]	Acute: randomized controlled pilot	VL30: *n* = 12 VL30: *n* = 12^b^	24/0	24 ± 1	180 ± 6	78 ± 8	Physically active sports science students; > 2; 1.0 ± 0.2; free weight bench press
González-García et al. (2020) [63]	Acute: randomized crossover design	VL20_OL_: *n* = 11 VL20_80%RM_: *n* = 11	10/1	25 ± 4	176 ± 8	77 ± 9	Unclear; unclear; 1.8 ± 0.3; Smith machine full back-squat
Held et al. (2021) [66]	Chronic: randomly assigned	VL10: *n* = 11	9/2^a^	20 ± 2	184 ± 5	76 ± 9	Highly trained rowers; ≥ 2; 1.7 ± 0.2, 2.2 ± 0.4, 1.5 ± 0.2 and 1.3 ± 0.2; free-weight back-squat, free-weight bench row, free-weight deadlift, and free-weight bench press, respectively
Krzysztofik et al. (2021) [68]	Acute: randomized crossover design	VL10: *n* = 16	0/16	24 ± 5	170 ± 6	64 ± 5	Resistance-trained amateur female volleyball players; 3 ± 4; 1.5 ± 0.2; free-weight back-squat
Martinez-Canton et al. (2020) [61]	Chronic: randomly assigned	VL20: *n* = 12 VL40: *n* = 10	22/0	23 ± 2	176 ± 6	76 ± 7	Physically active sports science students; 1.5 ± 4; 1.4 ± 0.2; Smith machine full back-squat
Muñoz-López et al. (2021) [80]	Acute: randomized crossover design	VL20: *n* = 30 VL40: *n* = 30	30/0	22 ± 2	176 ± 7	74 ± 11	Healthy individuals; unclear; 1.5 ± 0.2; Smith machine full back-squat
Nájera-Ferrer et al. (2021) [94]	Acute: randomized crossover design	VL20: *n* = 16 VL40: *n* = 16	16/0	36 ± 10	176 ± 7	77 ± 8	Resistance- and endurance-trained individuals; 2–5; 1.4 ± 0.3; Smith machine full back-squat
Nilo Dos Santos et al. (2021) [70]	Acute: randomized crossover design	VL20: *n* = 12	0/12	25 ± 5	163 ± 6	59 ± 11	Young women; 4.5 ± 4.2; 10RM = 46.2 ± 13.8; Smith machine parallel back-squat
Pareja-Blanco et al. (2017) [18]	Chronic: randomly assigned	VL20: *n* = 12 VL40: *n* = 10	22/0	23 ± 2	176 ± 6	76 ± 7	Physically active sports science students; 1.5 ± 4; 1.4 ± 0.2; Smith machine full back-squat
Pareja-Blanco et al. (2017) [76]	Chronic: randomly assigned	VL15: *n* = 8 VL30: *n* = 8	16/0	24 ± 4	174 ± 7	76 ± 9	Professional soccer players; unclear; 1.3 ± 0.3; Smith machine full back-squat
Pareja-Blanco et al. (2019) [22]	Acute: randomized crossover design	VL20_60%RM_: *n* = 17 VL40_60%RM_: *n* = 17 VL20_80%RM_: *n* = 17 VL40_80%RM_: *n* = 17	17/0	24 ± 4	180 ± 10	76 ± 11	Physically active sports science students; 3 ± 1.5; 1.4 ± 0.2; Smith machine full back-squat
Pareja-Blanco et al. (2020) [25]	Chronic: randomly assigned	VL0: *n* = 15 VL15: *n* = 16 VL25: *n* = 15 VL50: *n* = 16	62/0	24 ± 4	175 ± 6	76 ± 10	Resistance trained individuals; ≥ 1.5; 0.9 ± 0.2; Smith machine bench press
Pareja-Blanco et al. (2020) [26]	Chronic: randomly assigned	VL0: *n* = 14 VL10: *n* = 14 VL20: *n* = 13 VL40: *n* = 14	55/0	24 ± 4	175 ± 6	76 ± 10	Resistance trained individuals; 1.5 ± 4; 0.9 ± 0.2; Smith machine full back-squat
Pearson et al. (2020) [81]	Acute: counterbalanced crossover design	VL10: *n* = 12 VL20: *n* = 12 VL30: *n* = 12	12/0	23 ± 2	180 ± 7	89 ± 13	Semi-professional rugby union athletes; ≥ 2; unclear; free-weight parallel back-squat
Pérez-Castilla et al. (2018) [28]	Chronic: randomly assigned	VL10: *n* = 10 VL20: *n* = 10	20/0	22 ± 3 22 ± 2	175 ± 6 177 ± 6	74 ± 17 80 ± 15	Physically active sports science students; ≥ 2; 2.1 ± 0.4; Smith machine countermovement jump
Riscart-López et al. (2021) [42]	Chronic: randomly assigned	VL20_LP_: *n* = 11 VL20_UP_: *n* = 10 VL20_RP_: *n* = 11 VL20_CP_: *n* = 11	43/0	24 ± 6 24 ± 5 22 ± 3 23 ± 5	178 ± 5 177 ± 7 172 ± 6 180 ± 4	73 ± 8 71 ± 8 67 ± 6 75 ± 9	Physically active sports science students; 1.5–4; 1.3 ± 0.3; Smith machine full back-squat
Rissanen et al. (2022) [74]	Chronic: reverse counterbalancing sequence	VL20_M_: *n* = 12 VL20_F_: *n* = 11 VL40_M_: *n* = 11 VL40_F_: *n* = 11	23/22	26 ± 4	184 ± 8 167 + 7 178 ± 6 165 + 7	82 ± 8 61 ± 5 82 ± 14 60 + 8	Physically active individuals; ≥ 1; 1.3 ± 0.3 and 0.8 ± 0.2; Smith machine full back-squat and Smith machine bench press
Rodiles-Guerrero et al. (2020) [27]	Chronic: randomly assigned	VL10: *n* = 15 VL20: *n* = 15 VL30: *n* = 15	45/0	23 ± 2	173 ± 5	73 ± 6	Physically active individuals; ≥ 1; 1.0 ± 0.2; weight stack machine bench press
Rodríguez-Rosell et al. (2018) [16]	Acute: randomized crossover design	VL10_SQ_: *n* = 11 VL20_SQ_: *n* = 11 VL30_SQ_: *n* = 11 VL45_SQ_: *n* = 11 VL10_BP_: *n* = 10 VL20 _BP_: *n* = 10 VL30 _BP_: *n* = 10 VL45 _BP_: *n* = 10	21/0	24 ± 4	178 ± 4	78 ± 15	Physically active sports science students; 2–4; 1.5 ± 0.2 and 1.1 ± 0.2; Smith machine full back-squat and Smith machine bench press
Rodríguez-Rosell et al. (2020) [20]	Acute: randomized crossover design	VL10: *n* = 11 VL20: *n* = 11 VL30: *n* = 11 VL45: *n* = 11	11/0	24 ± 4	177 ± 7	74 ± 12	Physically active sports science students; 2–4; 1.5 ± 0.2; Smith machine full back-squat
Rodríguez-Rosell et al. (2020) [43]	Chronic: randomly assigned	VL10: *n* = 12 VL30: *n* = 13	25/0	23 ± 3 22 ± 3	177 ± 8 176 ± 7	75 ± 10 74 ± 9	Physically active sports science students; 1–3; 1.3 ± 0.3; Smith machine full back-squat
Rodríguez-Rosell et al. (2021) [44]	Chronic: randomly assigned	VL10: *n* = 12 VL30: *n* = 12 VL45: *n* = 12	36/0	23 ± 4 22 ± 2 22 ± 3	176 ± 4 177 ± 7 172 ± 8	71 ± 5 74 ± 9 72 ± 10	Physically active sports science students; 1–3; 1.3 ± 0.2; Smith machine full back-squat
Rodríguez-Rosell et al. (2021) [45]	Chronic: randomly assigned	VL15_LP_: *n* = 16 VL15_UP_: *n* = 16	32/0	24 ± 4 22 ± 3	176 ± 6 178 ± 7	76 ± 9 76 ± 8	Healthy and physically active sports science students; 1–3; 1.3 ± 0.3; Smith machine full back-squat
Sánchez-Moreno et al. (2020) [79]	Chronic: randomly assigned	VL25: *n* = 15 VL50: *n* = 14	29/0	27 ± 6 25 ± 6	176 ± 6 176 ± 5	74 ± 5 74 ± 8	Strength-trained individuals; 2–4; 0.5 ± 0.1; prone-grip pull-up exercise
Sousa-Fortes et al. (2020) [64]	Acute: randomiszd crossover design	VL20: *n* = 12	7/5	25 ± 5	169 ± 8	74 ± 18	Trained individuals; ~ 3; 1.3 and 1.1; free-weight half-squat and free-weight bench press
Tsoukos et al. (2019) [71]	Acute: randomized crossover design	VL10; *n* = 10 VL30; *n* = 10	10/0	26 ± 7	182 ± 5	85 ± 13	Physically active individuals; ≥ 3; 1.3 ± 0.2; Smith machine bench press
Tsoukos et al. (2021) [72]	Acute: randomized crossover design	VL10: *n* = 11 VL30: *n* = 11	11/0	26 ± 6	183 ± 5	85 ± 13	Resistance trained individuals; ≥ 3; 1.3 ± 0.2; Smith machine bench press
Varela-Olalla et al. (2019) [65]	Acute: unclear	VL20–32; *n* = 5	4/1	23 ± 5	169 ± 7	72 ± 18	Spanish Olympic wrestlers; ≥ 1; 1.5 ± 0.5; free-weight bench press
Varela-Olalla et al. (2020) [73]	Acute: observational	VL20; *n* = 15	15/0	23 ± 2	175 ± 6	73 ± 8	Recreationally active individuals; unclear; 0.7 ± 0.1; Smith machine half back-squat
Weakley et al. (2020) [21]	Acute: randomized crossover design	VL10; *n* = 12 VL20; *n* = 12 VL30; *n* = 12	12/0	23 ± 3	179 ± 6	87 ± 12	Team sport athletes from a British University and Colleges Super Rugby Club; ≥ 2; unclear; free weight back-squat
Weakley et al. (2020) [62]	Acute: randomized crossover design	VL10; *n* = 12 VL20; *n* = 12 VL30; *n* = 12	16/0	23 ± 2	180 ± 7	89 ± 13	Team sport athletes from a British University and Colleges Super Rugby Club; ≥ 2; unclear; free weight back-squat

*BM* body mass, *n* number of participants, *RT* resistance training, *VG* velocity training group, *VL##* whereby ## refers to the velocity loss threshold used (e.g., VL20 is 20% velocity loss threshold). Subscripts after VL## (e.g., VL20_80%RM_) refer to the following: *BP* protocol performed with the bench press exercise, *CP* constant programming model, *F* female, *FS* fixed number of sets, *LP* linear programming model, *M* male, *OL* optimal load that maximized power production, *##%RM* whereby ## refers to the percentage of repetition maximum, *RP* reverse programming model, *SQ* protocol performed with the back-squat exercise, *UP* undulating programming model, *VS* variable number of sets

^a^Sex distribution of participants included in statistical analysis was not specified

^b^Percussion therapy

**Table 2:** Summary of the acute studies included in the review

Study	Velocity loss threshold used; number of sets; load; inter-set rest	Exercises; load prescription method	Velocity variable; reference repetition for velocity loss calculation; number of repetitions performed below the threshold before termination of the set	Outcomes (methods of assessment)
Banyard et al. (2019) [77]	20%; 4.2 ± 0.9 (until reaching a total of 25 reps); 80%1RM; 2	Free-weight full back-squat; 1RM percentage based	Mean velocity; velocity of the single repetition performed at 80%1RM in the warm-up; 1	Mean velocity (4 linear position transducers); average number of repetitions per set
García-Sillero et al. (2021) [69]	30%; 4; 70%1RM; 3 30%; 4; 70%1RM; 3	Free-weight bench press; 1RM percentage based	Mean velocity; fastest repetition; 1	Mean velocity (linear position transducer); average number of repetitions per set
González-García et al. (2020) [63]	20%; 2; optimal load (60.9 ± 5.9%1RM); unclear 20%; 2; 80%1RM; unclear	Smith machine half back-squat; 1RM percentage based	Mean velocity; fastest repetition; 1	Mean velocity (rotatory encoder); CMJ height (force platform); RPE (Borg CR-10 Scale); average number of repetitions per set
Muñoz-López et al. (2021) [80]	20%; 3; 63.3 ± 2.1% 1RM; 5 40%; 3; 63.3 ± 2.1% 1RM; 5	Smith machine full back-squat; generalized load-velocity relationship based	Mean propulsive velocity; fastest repetition obtained in the first 3 repetitions; 1	Mean propulsive velocity (linear encoder); average number of repetitions per set
Nájera-Ferrer et al. (2021) [94]	20%; 3; 60%1RM; 2^a^ 40%; 3; 60%1RM; 2^a^ 20%; 3; 60%1RM; 2^b^ 40%; 3; 60%1RM; 2^b^	Smith machine deep back-squat; 1RM-percentage based	Mean propulsive velocity; fastest repetition; 1	Mean propulsive velocity, mean propulsive velocity attained against the absolute load that elicited 1.00 m·s^−1^ (linear velocity transducer); blood lactate concentration (portable lactate analyser); CMJ height (infrared timing system); average number of repetitions per set
Nilo Dos Santos et al. (2021) [70]	20% (32 ± 7%); 4; 10RM; 2	Smith machine parallel back-squat; 1RM-percentage based	Mean propulsive velocity; fastest repetition obtained in the first 3 repetitions; 1	Mean propulsive velocity (linear velocity transducer); average number of repetitions per set; RPE (OMNI-RES effort scale); rating of discomfort (Borg CR-10 scale)
Pareja-Blanco et al. (2019) [22]	20%; 3; 60%1RM; 4 40%; 3; 60%1RM; 4 20%; 3; 80%1RM; 4 40%; 3; 80%1RM; 4	Smith machine full back-squat; generalized load-velocity relationship based	Mean propulsive velocity; fastest repetition; 1	Mean propulsive velocity, percent change in velocity loss against the load that elicited a 1 m·s^−1^ (linear velocity transducer); percent change in CMJ height loss (infrared timing system); percent change in running sprint time loss (photocells); average number of repetitions performed during the 3 sets
Pearson et al. (2020) [81]	10%; 5; ~ 70%1RM; 3 20%; 5; ~ 70%1RM; 3 30%; 5; ~ 70%1RM; 3	Free-weight parallel back-squat; generalized load-velocity relationship based	Mean velocity; velocity reference of 0.70 m·s^−1^; 1	Mean velocity (linear position transducer); average number of repetitions per set
Rodríguez-Rosell et al. (2018) [16]	10%; 3; 50%1RM; 4	Smith machine full back-squat; generalized load-velocity relationship based	Mean propulsive velocity; fastest repetition; 1	Mean propulsive velocity, percent change in velocity loss against the load that elicited a 1 m·s^−1^ (linear velocity transducer); blood lactate concentration (portable lactate analyzer); average number of repetitions performed during the 3 sets
	10%; 3; 60%1RM; 4			
	10%; 3; 70%1RM; 4			
	10%; 3; 80%1RM; 4			
	20%; 3; 50%1RM; 4			
	20%; 3; 60%1RM; 4			
	20%; 3; 70%1RM; 4			
	20%; 3; 80%1RM; 4			
	30%; 3; 50%1RM; 4			
	30%; 3; 60%1RM; 4			
	30%; 3; 70%1RM; 4			
	30%; 3; 80%1RM; 4			
	45%; 3; 50%1RM; 4			
	45%; 3; 60%1RM; 4			
	45%; 3; 70%1RM; 4			
	45%; 3; 80%1RM; 4			
	15%; 3; 50%1RM; 4	Smith machine bench press; generalized load-velocity relationship based		
	15%; 3; 60%1RM; 4			
	15%; 3; 70%1RM; 4			
	15%; 3; 80%1RM; 4			
	25%; 3; 50%1RM; 4			
	25%; 3; 60%1RM; 4			
	25%; 3; 70%1RM; 4			
	25%; 3; 80%1RM; 4			
	40%; 3; 50%1RM; 4			
	40%; 3; 60%1RM; 4			
	40%; 3; 70%1RM; 4			
	40%; 3; 80%1RM; 4			
	55%; 3; 50%1RM; 4			
	55%; 3; 60%1RM; 4			
	55%; 3; 70%1RM; 4			
	55%; 3; 80%1RM; 4			
Rodríguez-Rosell et al. (2020) [20]	10%; 3; 50%1RM; 4 10%; 3; 60%1RM; 4 10%; 3; 70%1RM; 4 10%; 3; 80%1RM; 4 20%; 3; 50%1RM; 4 20%; 3; 60%1RM; 4 20%; 3; 70%1RM; 4 20%; 3; 80%1RM; 4 30%; 3; 50%1RM; 4 30%; 3; 60%1RM; 4 30%; 3; 70%1RM; 4 30%; 3; 80%1RM; 4 45%; 3; 50%1RM; 4 45%; 3; 60%1RM; 4 45%; 3; 70%1RM; 4 45%; 3; 80%1RM; 4	Smith machine full back-squat; generalized load-velocity relationship based	Mean propulsive velocity; fastest repetition; 1	Mean propulsive velocity, percent change in velocity loss against the load that elicited a 1 m·s^−1^ (linear position transducer), blood lactate concentration (portable lactate analyzer); percent change in CMJ height (infrared timing system)
Sousa-Fortes et al. (2020) [64]	20%, 5; 15RM; 3:20 min	Free-weight half back-squat and bench press; 1RM-percentage based	Mean velocity; unclear; 1	Mean velocity (linear position transducer); average number of repetitions per set
Tsoukos et al. (2019) [71]	10%; 40%1RM; 1; 0 10%; 60%1RM; 1; 0 30%; 40%1RM; 1; 0 30%; 60%1RM; 1; 0	Smith machine bench press throw; 1RM percentage based	Mean velocity; fastest repetition; 1	Mean propulsive velocity (linear position transducer); average number of repetitions per set
Tsoukos et al. (2021) [72]	10%; 1; 80%1RM; 0 30%; 1; 80%1RM; 0	Smith machine bench press; 1RM-percentage based	Mean velocity; fastest repetition; 1	Mean velocity (linear position transducer); average number of repetitions per set
Varela-Olalla et al. (2019) [65]	20–27.3%; 1; 40–45%1RM; 0 22.1–29.4%; 1; 55–60%1RM; 0 20.7–31.1%; 1; 70–75%1RM; 0	Free-weight bench press; generalized load-velocity relationship based	Mean velocity; fastest repetition; 2	Mean velocity (linear position transducer); average number of repetitions per set; RPE (OMNI-RES scale)
Varela-Olalla et al. (2020) [73]	20%; 1; ~ 85%1RM; 0	Smith machine half squat; generalized load-velocity relationship based	Mean propulsive velocity; fastest repetition; 1	Mean propulsive velocity (linear position transducer); blood lactate concentration (portable lactate analyser); CMJ height (smartphone app)
Weakley et al. (2020) [21]	10%; 5; ~ 70%1RM; 3 20%; 5; ~ 70%1RM; 3 30%; 5; ~ 70%1RM; 3	Free-weight parallel back-squat; generalized load-velocity relationship based	Mean velocity; velocity reference of 0.70 m·s^−1^;1	Blood lactate concentration (portable lactate analyser); CMJ (force plate); average number of repetitions per set; differential-RPE of the lower peripheries and the breathlessness (verbal anchors on the CR100 scale)
Weakley et al. (2020) [62]	10%; 5; ~70%1RM; 3 20%; 5; ~ 70%1RM; 3 30%; 5; ~ 70%1RM; 3	Free-weight parallel back-squat; generalized load-velocity relationshi -based	Mean velocity; velocity reference of 0.70 m·s^−1^;1	Mean velocity (linear position transducer); average number of repetitions per set

*1RM* one-repetition maximum, *CMJ* countermovement jump, *RPE* rate of perceived effort

^a^Endurance training followed by resistance training

^b^Resistance training followed by endurance training

Note: only outcomes of interest were reported in this table; for a more extended version, see ESM

**Table 3:** Summary of the longitudinal studies included in the review

Study	Training protocol (duration in weeks; sessions/w; exercise; loads)	Velocity loss threshold; number of sets; inter-set rest	Velocity loss used for all exercises?	Adherence	Outcomes (methods of assessment)	Comparisons between the groups (outcomes)
Alcazar et al. (2021) [60]	8; 2; Smith machine full back-squat; from 70%1RM to 85%1RM	**VL0**; 3; 4 **VL10**; 3; 4 **VL20**; 3; 4 **VL40**; 3; 4	Yes	100%	1RM, *F*_0_, *v*_0_*,* *P*_max_, a, and *a*/*F*_0_ (2 force plates synchronized with a linear velocity transducer)	**VL0 = VL10 = VL20 = VL40** (L-*F*_0_, L-*v*_0_*,* L-*P*_max_, L-*a*, H-*F*_0_, H-*v*_0_*,* H-P_max_, and H-*a/F*_0_ **VL0 and VL10 > VL40** (H-*v*_0_))
Andersen et al. (2021) [29]	9; 2; leg press and leg extension; 85%1RM (leg press) and 75%RM (leg extension)	**VL15**; 4 (sessions 1–2) and 6 (sessions 3–9) for the leg press and 4 (sessions 1–5) and 6 (sessions 6–9) for leg extension; 2.5 **VL30**; 2 (sessions 1–2) and 3 (sessions 3–9) for the leg press and 2 (sessions 1–5) and 3 (sessions 6–9) for leg extension; 2.5	No	96.1%	1RM, mean velocity attained at 30%1RM (MV_30%1RM_), 45%1RM (MV_45%1RM_), 60%1RM (MV_60%1RM_), 75%1RM (MV_75%1RM_), mean power attained at 30%1RM (MP_30%1RM_), 45%1RM (MP_45%1RM_), 60%1RM (MP_60%1RM_), 75%1RM (MP_75%1RM_), as well as *L*_0_ and *v*_0_ obtained from the load-velocity relationship (linear encoder) MVC, RFD for the period between 20 and 80% of MVC (RFD_20–80%MVC_) in addition to 50 ms (RFD_50ms_), 100 ms (RFD_100ms_), and 200 ms (RFD_200ms_)—(force platform) Thickness and architecture of VL and RF (B-mode ultrasound)	**VL15 = VL30** (1RM, MV_30%1RM_, MV_45%1RM_, MV_60%1RM_, MV_75%1RM_, MP_30%1RM_, MP_45%1RM_, MP_60%1RM_, MP_75%1RM_, *L*_0_, *v*_0_, MVC, RFD_20–80%MVC_, RFD_50ms_, RFD_100ms_ and RFD_200ms_; VL, RF, PA, and FL)
Dorrell et al. (2020) [75]	6; 2; back squat, bench press, strict overhead press (only sessions 1, 3, 5, 7, 9, 11, and 12), and deadlift (only sessions 2, 4, 6, 8, 10, 11, and 12); from 70%1RM to 95%1RM	**VL20** (below the target velocity of each specific zone); 3; unclear	No	100%	1RM (linear position transducer) CMJ height (jump mat)	**VL20:** back squat 1RM (↑), bench press 1RM (↑) strict overhead press 1RM (↑), deadlift 1RM (↑), CMJ height (↑)
Fernandez-Ortega et al. (2020) [67]	12; 3; Smith machine full back-squat and cycle ergometer; 65%1RM (0.70 m·s^−1^) [squat] and 65% of the load applied in the initial assessment (5.3% of body weight) [cycle ergometer]	**VL20** (squat) RPML20 (cycle ergometer); 4; 3	No	Unclear	CMJ and SJ height (infrared timer system) *T*_0–30_ (infrared-light photocell system) 1RM Maximum power (absolute [*P*_max_-C] and relative [*P*_max_-RC]) and velocity (*V*_max_-C) on the cycloergometer (Wingate test) Maximal power (*P*_max_-S) and velocity (*V*_max_-S) in the squat (squat test with loads of 30, 40, 45, 60, 70, and 80%1RM) (linear velocity transducer)	**VL20:** *T*_0–30_ (↑), 1RM (↑), CMJ height (↑), SJ height (↑), *P*_max_-C (↑), *P*_max_-RC (↑), *V*_max_-C (↑), *P*_max_-S (↑), *V*_max_-S (↑)
Galiano et al. (2020) [78]	7; 2; Smith machine full back-squat; ~ 1.14 ± 0.03 m·s^−1^ (~ 50%1RM)	**VL5**; 3; 3 **VL20**; 3; 3	Yes	100%	1RM, AV, and AV ≥ 1 (linear velocity transducer) T_0-20_ (photocells) CMJ height (infrared timing system)	**VL5 = VL20** (1RM, AV, AV ≥ 1, AV < 1, T20, and CMJ height)
Held et al. (2021) [66]	8; 2; power clean, squat, bench row, deadlift, and bench press; 80%1RM	**VL10**; 4; 2–3	No	~ 94%	1RM	**VL10:** squat 1RM (↑), bench row 1RM (↑), deadlift 1RM (↑), bench press 1RM (↑), and 1RM_total_ (↑)
Martinez-Canton et al. (2020) [61]	8; 2; Smith machine full back-squat; from 0.82 m·s^−1^ (~ 70%1RM) to 0.60 m·s^−1^ (~ 85%1RM)	**VL20**; 3; 4 **VL40** (from VL20 to VL50); 3; 4	Yes	100%	Fatigue test (as many repetitions as possible against 60%1RM load until the velocity felt below 0.50 m·s^−1^), FT-MNR, and FT-AV (linear velocity transducer)	
Pareja-Blanco et al. (2017) [18]	8; 2; Smith machine full back-squat; from 0.82 m·s^−1^ (~ 70%1RM) to 0.60 m·s^−1^ (~ 85%1RM)	**VL20**; 3; 4 **VL40** (from 20 to 50%); 3; 4	Yes	100%	1RM, AV, AV > 1, and AV < 1 (linear velocity transducer) *T*_0–20_ (photocells) CMJ height (infrared timing system) Muscle volume of QF, RF, VM and VL + VI (1.5-T scanner) Muscle CSA (1.5-T scanner) Fiber CSA, CSA-I, CSA-IIA, CSA-IIAX, and CSA-IIX (muscle biopsy)	**VL20 = VL40** (1RM, AV < 1, T20, QF, RF, and VM) **VL20 > VL40** (AV, AV > 1, CMJ height, CSA, CSA-I, CSA-IIA, CSA-IIAX, and CSA-IIX) **VL40 > VL20** (VL, VI)
Pareja-Blanco et al. [76] (2017)	6; 3; Smith machine full back-squat; from ~ 1.13 m·s^−1^ (~ 50%1RM) to ~ 0.82 m·s^−1^ (~ 70%1RM)	**VL15**; 2 (sessions 1, 4, 7, 11, 15, and 18) or 3 (sessions 2, 3, 4, 6, 8, 9, 10, 12, 13, 14, 16, and 17); 4 **VL30**; 2 (sessions 1, 4, 7, 11, 15, and 18) or 3 (sessions 2, 3, 4, 6, 8, 9, 10, 12, 13, 14, 16, and 17); 4	No	85%	1RM and AV (linear velocity transducer) YIRT *T*_0–30_ (photocells) CMJ height (infrared timing system)	**VL15 = VL30** (1RM, AV, YIRT, and T_0–30_) **VL15 > VL30** (CMJ height)
Pareja-Blanco et al. (2020) [25]	8; 2; Smith machine bench press; from 0.65 ± 0.07 m·s^−1^ (70%1RM) to 0.41 ± 0.05 m·s^−1^ (85%1RM)	**VL0**; 3; 4 **VL15**; 3; 4 **VL25**; 3; 4 **VL50**; 3; 4	Yes	100%	MIF, RFD_max_, slope of the force–time curve obtained over 50 ms (RFD_0–50_), 100 ms (RFD_0–100_), 150 ms (RFD_0–150_), 200 ms (RFD_0–200_), and 400 ms (RFD_0–400_)—(dynamometric platform) 1RM, *v*_0_ (bar weight < 0.2 kg), AV, AV > 0.8, and AV < 0.8 (linear velocity transducer) Fatigue test (as many repetitions as possible against 70%1RM load until the muscle failure), FT-MNR, and FT-AV (linear velocity transducer) Muscle CSA of PM (B-mode ultrasonography)	**VL0 = VL15 = VL25 = VL50** (MIF, RFD_max_, RFD_0–50_, RFD_0–100_, RFD_0–150_, RFD_0–200_, RFD_0–400_, 1RM, *v*_0_, AV, AV > 0.8, AV < 0.8, FT-MNR, FT-AV, and PM) **VL50 > VL0** (PM)
Pareja-Blanco et al. (2020) [26]	8; 2; Smith machine full back-squat; from 70%1RM to 85%1RM	**VL0**; 3; 4 **VL10**; 3; 4 **VL20**; 3; 4 **VL40**; 3; 4	Yes	100%	*T*_0–10_, *T*_10–20_, and *T*_0–20_ (photocells) CMJ height (infrared timing system) MVIC, RFD_max_, RFD_0–50_, RFD_0–100_, and RFD_0–150_ (dynamometric platform) 1RM, AV, AV > 1, and AV < 1 (linear velocity transducer) Fatigue test (as many repetitions as possible against 70%1RM load until the velocity fell below 0.5 m·s^−1^), and FT-MNR (linear velocity transducer) Muscle CSA and architecture of VL (B-mode ultrasonography)	**VL0 = VL10 = VL20 = VL40** (*T*_0–10_, *T*_10–20_, *T*_0–20_, CMJ, MVIC, RFD_max_, RFD_0-50_, RFD_0–100_, RFD_0–150_, 1RM, AV, AV > 1, AV < 1, FT-MNR, CSA, PA, and FL) **VL0 > VL10**
Pérez-Castilla et al. (2018) [28]	4; 2; Smith machine countermovement jump; 1.20 m·s^−1^ (~ 40%1RM)	**VL10**; the number of sets was extended until completing 36 repetitions; 4 **VL20**; the number of sets was extended until completing 36 repetitions; 4	No	100%	CMJ height, *F*_0_, *v*_0_, *P*_max_, and *a* (infrared platform) 1RM, MPV attained at 20 (MPV_20_), 40 (MPV_40_), 60 (MPV_60_), and 80 (MPV_80_) kg *T*_0–15_ (photocells)	**VL10 = VL20** (*F*_0_, *v*_0_, *a*, P_max_, 1RM, MPV_20_, MPV_40_, MPV_60_, MPV_80_, CMJ height, and *T*_0–15_)
Riscart-López et al. (2021) [42]	8; 2; Smith machine full back-squat; from 50%1RM to 85%1RM with increments of 5%1RM every 2 sessions (LP), from 85%1RM to 50%1RM with decreases of 5%1RM every 2 sessions (RP), from 50%1RM to 85%1RM with changes in %1RM every session (UP), and ~ 67.5%1RM (CP)	**VL20**; 3; 4	Yes	100%	1RM, AV, AV > 1, and AV < 1 (linear velocity transducer) T_0–20_ (photocells) CMJ height (infrared timing system)	**LP = RP = UP = CP** (1RM, AV, AV > 1, AV > 1, T_0–20_, and CMJ height)
Rissanen et al. (2022) [74]	8; 2; Smith machine full back-squat and Smith machine bench press; from 65%1RM to 75%1RM	**VL20 male**; 2 (session 1), 3 sessions 2–3–6–7–11), 4 (sessions 4–8–12–13) and 5 (sessions 5–9–10–14–15); 3 **VL20 female**; 2 (session 1), 3 (sessions 2–3-6–7-11), 4 (sessions 4–8–12–13) and 5 (sessions 5–9–10–14–15); 3 **VL40 male**; 2 (session 1), 3 sessions 2–3-6–7-11), 4 (sessions 4–8-12–13) and 5 (sessions 5–9-10–14-15); 3 **VL40 female**; 2 (session 1), 3 sessions 2–3–6–7–11), 4 (sessions 4–8–12–13) and 5 (sessions 5–9–10–14–15); 3	No	**VL20 male**; 98 ± 3% **VL20 female**; 95 ± 6% **VL40 male**; 97 ± 5% **VL40 female**; 95 ± 4%	1RM, “low” MPV values (< 70%1RM) and “high” MPV values (> 70%1RM) [linear velocity transducer] CMJ height (force platform) Muscle CSA of vastus lateralis (B-mode ultrasound)	**VL20 male = VL20 female = VL40 male = VL40 female** (Smith machine full back-squat and bench press 1RM, Smith machine full back-squat and bench press “low” and “high” MPV values, CMJ height, vastus lateralis CSA)
Rodiles-Guerrero et al. (2020) [27]	5; 3; weight stack machine bench press; from 0.67 m·s^−1^ (~ 65%1RM) to 0.39 m·s^−1^ (~ 85%1RM)	**VL10**; 4; 3 **VL30**; 4; 3 **VL50**; 4; 3	Yes	Unclear	1RM, AV, AV ≥ 0.8, and AV < 0.8 (linear velocity transducer)	**VL10 = VL30 = VL50** (1RM, AV, AV ≥ 0.8, and AV < 0.8)
Rodríguez-Rosell et al. (2020) [43]	8; 2; Smith machine full back-squat; from ~ 0.84 m·s^−1^ (~ 70%1RM) to ~ 0.60 m·s^−1^ (~ 85%1RM)	**VL10**; 3; 4 **VL30**; 3; 4	Yes	100%	1RM, AV, AV > 1, AV < 1, MPV attained against 30 kg (MPV_30_), MPV_40_, 50 kg (MPV_50_), MPV_60_, 70 kg (MPV_70_), and MPV_80_ (linear velocity transducer) *T*_0–10_ and *T*_0–20_ (photocells) CMJ height (infrared timing system) Fatigue test (as many repetitions as possible against an absolute load move to ~ 0.84 m·s^−1^ (~ 70%1RM until the MPV fell below 0.5 m·s^−1^), and FT-MNR (linear velocity transducer)	**VL10 = VL30** (1RM, AV, AV > 1, AV < 1; MPV_30_, MPV_40_, MPV_50_, MPV_60_, MPV_70_, MPV_80_, CMJ height, and FT-MNR) **VL10 > VL30** (*T*_0–10_, and *T*_0–20_)
Rodríguez-Rosell et al. (2021) [45]	8; 2; Smith machine full back-squat; from ~ 1.16 m·s^−1^ (~ 50%1RM) to ~ 0.68 m·s^−1^ (~ 80%1RM) using LP and UP	**VL15**; 3; 4	Yes	100%	1RM, AV, AV > 1, and AV < 1 (linear velocity transducer) CMJ height (infrared timing system) Fatigue test (as many repetitions as possible against ~ 1.16 m·s^−1^ (~ 60%1RM) load until the MPV fell below 0.5 m·s^−1^), and FT-MNR (linear velocity transducer)	**LP > UP** (1RM, AV, AV > 1, AV < 1, and FT-MNR) **LP = UP** (CMJ height)
Rodríguez-Rosell et al. (2021) [44]	8; 2; Smith machine full back-squat; from ~ 1.08 m·s^−1^ (~ 55%1RM) to ~ 0.84 m·s^−1^ (~ 70%1RM)	**VL10**; 3; 4 **VL30** (from VL20 to VL30); 3; 4 **VL45** (from VL20 to VL45); 3; 4	Yes	100%	1RM, AV, AV > 1, AV < 1, MPV_30_, MPV_40_, MPV_50_, MPV_60_, MPV_60_, and MPV_80_ (linear velocity transducer) *T*_0–10_ and *T*_0–20_ (photocells timing gates) CMJ height (infrared timing system) Fatigue test (as many repetitions as possible against ~ 0.84 m·s^−1^ (~ 70%1RM) load until the MPV fell below 0.5 m·s^−1^), and FT-MNR (linear velocity transducer)	**VL10 > VL30 and VL45** (CMJ height, AV, and AV > 1) **VL30 = VL45** (CMJ height, AV, and AV > 1) **VL10 = VL45 = VL30** (*T*_0–10_, *T*_0–20_, 1RM, AV < 1, MPV_30_, MPV_40_, MPV_50_, MPV_60_, MPV_70_, MPV_80_, and FT-MNR)
Sánchez-Moreno et al. (2020) [79]	8; 2; prone-grip pull-up; body mass	**VL25**; 2 (sessions 1–3 and 16), 3 (sessions 4–8 and 15) or 4 (sessions 9–14); 3 **VL50**; 2 (sessions 1–3 and 16), 3 (sessions 4–8 and 15) or 4 (sessions 9–14); 3	Yes	95%	1RM, AV, and MPV_best_ (linear velocity transducer) Fatigue test to failure, FT-MNR and FT-AV (linear velocity transducer)	**VL25 > VL50** (1RM, AV, and MPV_best_, and FT-AV) **VL25 = VL50** (FT-MNR)

*1RM* one-repetition maximum, *a* slope of the force–velocity relationship, *a*/*F*_0_, curvature of the force–velocity relationship, *AV* average velocity attained against all absolute loads common to pre-test and post-test, *AV* > *0.8* average velocity attained against absolute loads that were lifted faster than 0.8 m·s^−1^, *AV* < *0.8* average velocity attained against absolute loads that were lifted slower than 0.8 m·s^−1^, *AV* ≥ *1* average velocity attained for absolute loads moved at velocities equal to or faster than 1 m·s^−1^, *AV* < *1* the average velocity attained for absolute loads moved slower than 1 m·s^−1^, *CP* constant programming, *CSA* cross-sectional area, *F*_0_ maximal force, *FL* fascicle length, *FT-AV* average velocity attained against the same number of repetitions during the fatigue test, *FT-MNR* maximum number of repetitions during the fatigue test, *L*_0_ maximal load, *LP* linear programming, *MIF* maximal isometric force, *MPV* mean propulsive velocity, *MPV*_*best*_ the fastest MPV attained without additional weight, *MVC* maximal voluntary contraction, *PA* pennation angle, *PM* pectoralis major, *P*_*max*_ maximal force, *QF* quadriceps femoris, *RF* rectus femoris, *RFD*_*max*_ maximal rate of force development, *RIR* reps in reserve, *RP* reverse programming, *T*_0*–1*0_ 10-sprint time, *T*_0*–2*0_ 20-sprint time, *T*_0*–3*0_ 30-sprint time, *T*_*1*0*–2*0_ time to cover 10–20 m, *T*_0*–15*_ 15-sprint time, *UP* undulating programming, *v*_0_ maximal velocity, *VL* vastus lateralis, *VL##* whereby ## refers to the velocity loss threshold used (e.g., VL20 is a 20% velocity loss threshold), *VL* + *VI* vastus lateralis and vastus intermedius, *VM* vastus medialis, *YIRT* total distance covered in the Yo-Yo Intermittent Recovery Test level 1, ↑ reflects an improvement in performance, ⟷ no significant change, and ↓ a decrease in performance. Andersen et al. [29] did not provide statistical inferences for pre-post changes per group. Therefore, ⟷ reflects a < moderate effect size and ↑ reflects a > moderate effect size. Held et al. [66], Fernandez Ortega et al. [67], and Dorrell et al. [75] evaluated only one VL threshold. Therefore, group differences column for these studies represents pre-post changes

Note: only outcomes of interest were reported in this table; for a more extended version, see ESM

### Risk of Bias Assessment

**Fig. 2:**
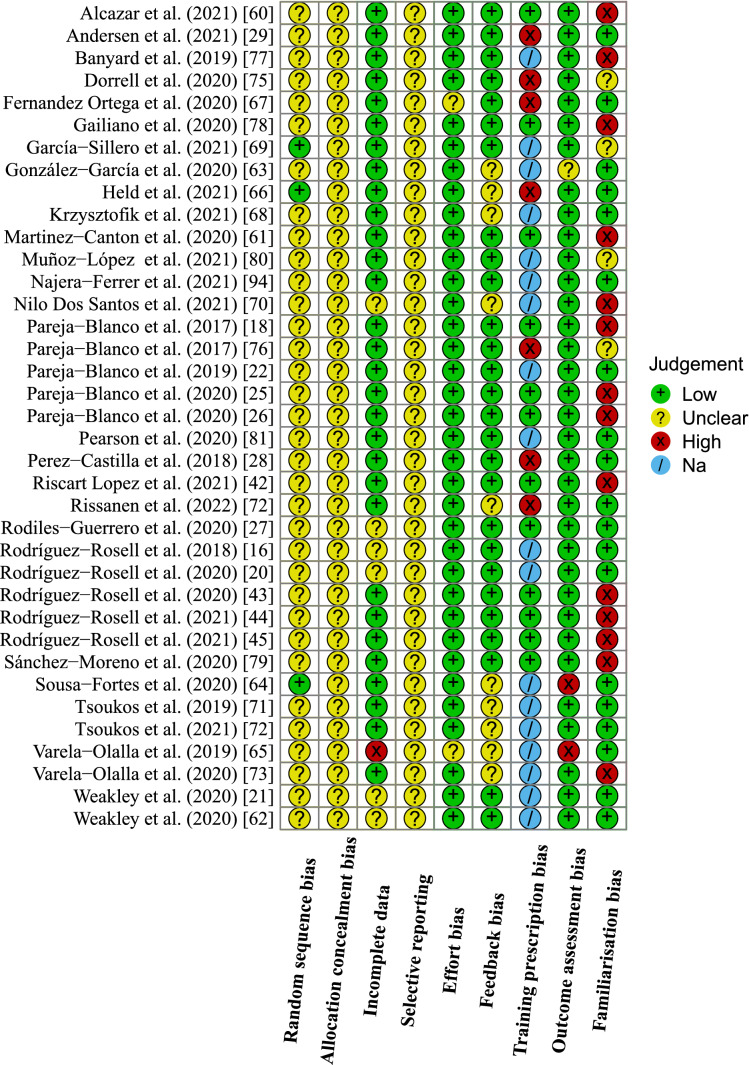
Risk of bias assessment for all included studies. *Na* not applicable

Only three studies [64, 66, 69] provided sufficient information regarding the method of randomization and were therefore at a low risk of an order effect bias. The remaining studies were classified as an unclear risk as they did not provide sufficient information regarding the method of randomization. No studies provided information regarding allocation concealment. One study [65] was at a high risk of attrition bias, excluding randomized participants (or their data) from the analysis without sufficient reason. Six studies [16, 20, 21, 43, 62, 70] did not provide sufficient information on the number of participants assessed and included in the analysis after reporting that some of them did not complete the entire intervention or all procedures and hence, had an unclear risk of attrition bias. No studies pre-registered their protocols on a publicly available registry platform, thus it was unclear whether selective reporting bias was present. Two studies [65, 67] had an unclear risk of effort bias as they did not provide information regarding the instructions to perform the concentric actions as fast as possible. The remaining studies had a low risk of effort bias as the instruction to perform concentric actions as fast as possible was given. Ten studies [63–66, 68, 70–74] did not provide any information on the provision of velocity feedback and hence, had an unclear risk of feedback bias. The rest of the studies either provided feedback to all groups or standardized the conditions between groups by not providing any feedback. Seven studies [28, 29, 66, 67, 74–76] were at a high risk of training prescription bias because the participants performed other forms of training (additional non-standardized RT, endurance training, or playing sports), or because not all exercises used VL thresholds, but rather a combination of training prescriptions. Two studies [64, 65] used a linear encoder that was not, to our knowledge, validated in the peer-reviewed literature whereas all other studies used valid and reliable methods, equipment, or instruments to evaluate their outcomes of interest. Fourteen studies [18, 25, 26, 42–45, 60, 61, 70, 73, 77–79] were at a high risk of bias for not having a familiarization session. Four studies [69, 75, 76, 80] did not provide sufficient information regarding their familiarization sessions and hence, had an unclear risk of bias The rest of the studies provided sufficient information about familiarization session procedures or specifically stated that all participants were accustomed to the study protocols (i.e., performed them in the past). The risk of bias assessment is also illustrated in Fig. 2.

### Acute Studies

**Fig. 3:**
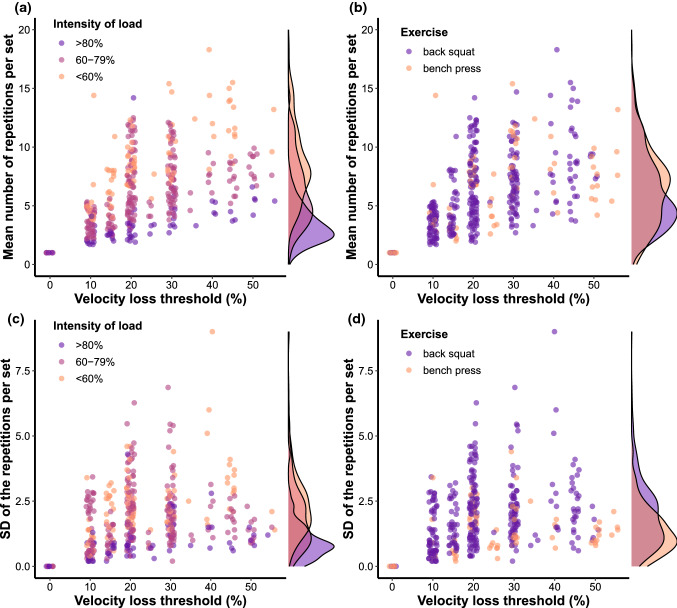
Visual representation of the mean number of repetitions performed per set by intensity of load (**a**) and exercise (**b**), as well as standard deviation of the number of repetitions performed per set by intensity of load (**c**) and exercise (**d**) across the velocity loss thresholds reported in the literature. Note, longitudinal studies were also included here when they reported number of repetitions per set for each training session. Note, one study outlier was removed from the figure as the participants completed more than 25 repetitions in a set

**Fig. 4:**
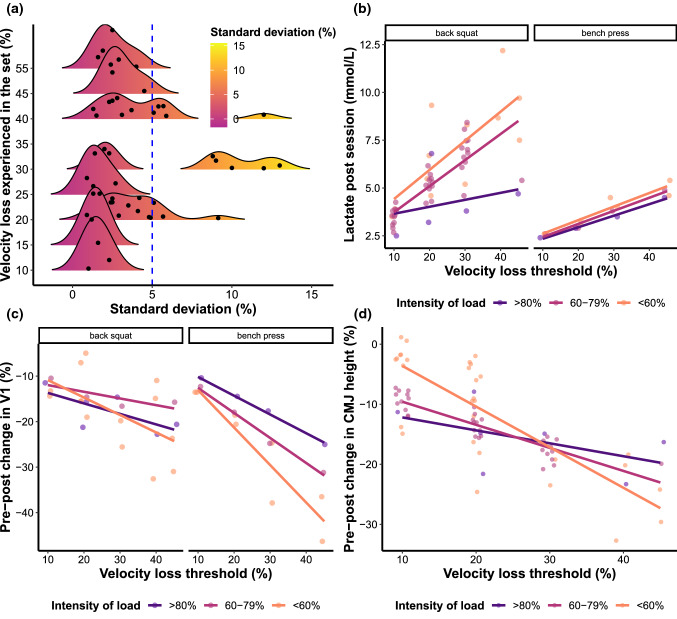
Visual representation of the variability of the actual velocity loss experienced in a set (**a**), post-session blood lactate accumulation across velocity loss thresholds by exercise and intensity of load (**b**), pre-post percent change in velocity against the load that can be lifted at 1 m·s^−1^ (V1) by exercise and intensity of load (**c**), and pre-post percent change in countermovement jump (CMJ) height (**d**) across velocity loss thresholds reported in the literature

The following variables were visualized: (1) the mean and standard deviation of the number of repetitions performed in the set; (2) changes in countermovement jump height performance; (3) velocity against the load that can be lifted at 1 m·s^−1^ in a rested state (V1); and (4) blood lactate concentration after training sets or the entire session (Figs. 3, 4). In addition, to examine the discrepancy between the VL threshold prescribed and the actual VL experienced by the participants in each study, standard deviations of the actual VL experienced were visually represented using density plots (Fig. 3).

### Longitudinal Studies

**Fig. 5:**
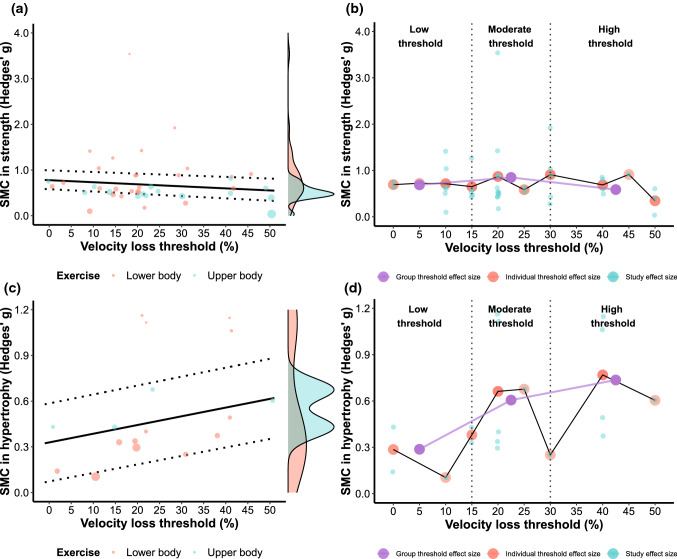
Multilevel mixed-effects meta-regression illustrating the effects of velocity loss thresholds on muscle strength gains (also see Table 4) after controlling for exercise, study duration, and strength levels of the individuals (**a**), and the effects of velocity loss thresholds on muscle hypertrophy (**c**). Dose–response relationship considering (1) individual study effect sizes (green circles); (2) average effect sizes of individual velocity loss thresholds (red circles); and (3) average effect sizes of low (≤ 15%), moderate (> 15% < 30%), and high (> 30%) grouped velocity loss thresholds (purple circles and lines) between velocity loss and muscle strength (**b**) and hypertrophy (**d**) gains. Black (non-vertical) solid and dotted lines represent estimated relationships and corresponding upper and lower 95% confidence intervals, whereas vertical dotted lines represent boundaries between velocity loss thresholds. S*MC* standardized mean change

**Fig. 6:**
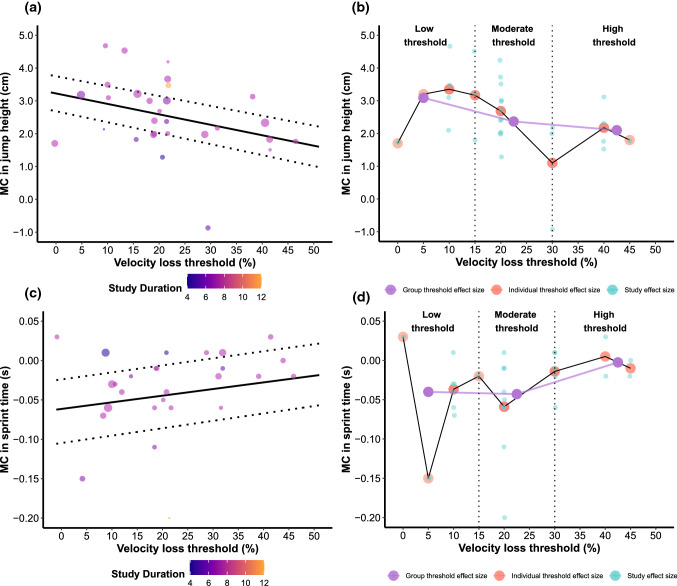
Multilevel mixed-effects meta-regression illustrating the effects of velocity loss thresholds on countermovement jump (**a**) and running sprint time (**c**) after controlling for study duration (also see Table 4). For (**a**) and (**c**), larger data points received greater weighting than smaller data points. Dose–response relationship considering (1) individual study effect sizes (green circles); (2) average effect sizes of individual velocity loss thresholds (red circles); and (3) average effect sizes of low (≤ 15%), moderate (> 15% < 30%), and high (> 30%) grouped velocity loss thresholds (purple circles and lines) between velocity loss and countermovement jump (**b**) and running sprint (**d**) performance improvement. Black, solid, and dotted (non-vertical) lines represent estimated relationships and corresponding upper and lower 95% confidence intervals, whereas vertical dotted lines represent boundaries between velocity loss thresholds. *MC* mean change

**Fig. 7:**
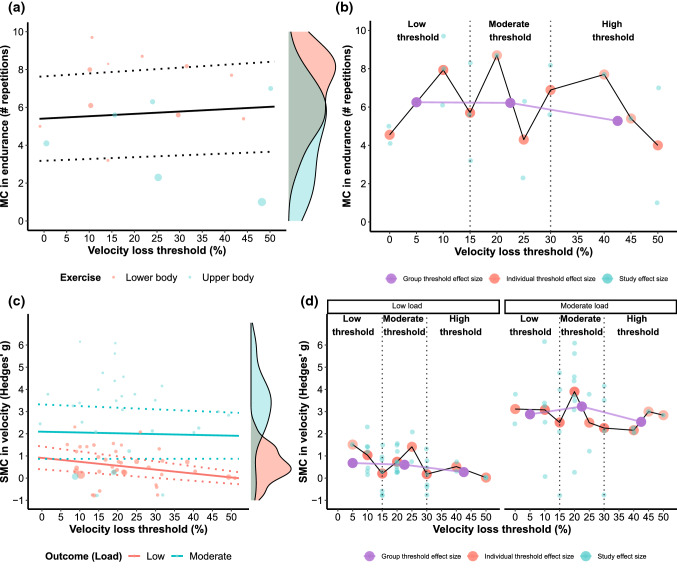
Multilevel mixed-effects meta-regression illustrating the effects of velocity loss thresholds on muscle endurance quantified by the number of repetitions performed in a fatigue test (**a**). Multivariate mixed-effects meta-regression illustrating the effects of velocity loss thresholds on velocity against low (> 1 m·s^−1^; red circles and lines), and moderate (< 0.8 m·s^−1^; green circles and lines) loads (**c**). For **a** and **c**, larger data points received greater weighting than smaller data points. Dose–response relationship considering (1) individual study effect sizes (green circles); (2) average effect sizes of individual velocity loss thresholds (red circles); and (3) average effect sizes of low (≤ 15%), moderate (> 15% < 30%), and high (> 30%) grouped velocity loss thresholds (purple circles and lines) between velocity loss and muscle endurance (**b**) and velocity against submaximal loads (**d**) performance improvement. Black, green, and red (solid and dotted) lines represent estimated relationships and corresponding upper and lower 95% confidence intervals, whereas vertical, dotted, and black lines represent boundaries between velocity loss thresholds. *MC* mean change, *SMC* standardized mean change

For all multilevel models, significant moderators and sensitivity analyses are described in the text, whereas their output is presented in Table 4 and visualized in Figs. 5, 6 and 7. For the multivariate model, all information is described in the text, and model estimates are visualized in Fig. 6b. Dose–response relationships, as quantified by effect sizes, between VL and outcomes of interest are also illustrated in Figs. 5, 6 and 7.

#### Muscle Strength

The final multilevel model investigating the effects of different VL thresholds on maximal strength gains revealed exercise, strength levels, and study duration to be significant moderators (Table 4; Fig. 5a). Two individual groups from two different studies were identified as influential. Excluding these influential groups from the analysis affected the interpretation of the model, with exercise (*b* = − 0.163 [− 0.416, 0.094]; *p* = 0.206) and strength levels (*b* = − 0.181 [− 0.655, 0.293]; *p* = 0.444) no longer being significant moderators.

#### Muscle Hypertrophy

The final multilevel model investigating the effects of different VL thresholds on muscle hypertrophy revealed VL to be a significant moderator (Table 4; Fig. 5c). Two individual groups from two studies were identified as influential. Excluding these influential groups from the analysis affected the interpretation of the model, with VL no longer being a significant moderator (*b* = 0.005 [− 0.002, 0.013]; *p* = 0.144).

#### Muscle Endurance

The final multilevel model investigating the effects of different VL thresholds on muscle endurance did not reveal VL to be a significant moderator (Table 4; Fig. 7a). Two individual groups from two different studies were identified as influential. However, the overall results were robust to their exclusion from the model as the interpretation of the model did not change.

#### Countermovement Jump Height

The final multilevel model investigating the effects of different VL thresholds on the countermovement jump revealed VL and study duration to be significant moderators (Table 4; Fig. 6a). Three individual groups from three different studies were identified as influential. However, the overall results were robust to their exclusion from the model as the interpretation of the model did not change. In fact, the confidence in the estimate for both VL (*b* = − 0.048 [− 0.073, − 0.023]; *p* = 0.001) and study duration (*b* = 0.400 [0.105, 0.695]; *p* = 0.010) increased after their removal.

#### Sprint Time

The final multilevel model investigating the effects of different VL thresholds on sprint time revealed VL and study duration as significant moderators (Table 4; Fig. 6c). Three individual groups from three different studies were identified as influential. Excluding these influential groups from the analysis affected the interpretation of the model, with study duration no longer being a significant moderator (*b* = − 0.005 [− 0.031, 0.021]; *p* = 0.696).

**Table 4:** Moderator analyses (of multilevel models)

Outcome	Moderator	Studies (k)	Effect size (*n*)		*β* (95% CI)	*t* value	*p* value	Overall^a^	*R*^2^ (level 2)	*R*^2^ (level 3)	*I*^2^ (level 2)	*I*^2^ (level 3)
Maximal strength	Intercept^b^	17		41	0.785 (− 0.435, 2.006)	1.305	0.2	*F*(4, 36) = 6.14	100	49.28	0	65.38
	Velocity loss	17		41	− 0.004 (− 0.008, 0.001)	− 1.707	0.097					
	Upper body	17		41	− 0.371 (− 0.667, − 0.076)	− 2.546	0.015					
	Strength levels	17		41	− 0.606 (− 1.103, − 0.110)	− 2.476	0.018					
	Study duration	17		41	0.113 (0.030, 0.215)	2.257	0.03					
Muscle hypertrophy	Intercept	6		17	0.329 (0.050, 0.608)	2.612	0.024	*F*(1, 15) = 4.79	99.99	17.76	0	82.54
	Velocity loss	6		17	0.057 (0.001, 0.011)	2.187	0.045					
Muscle endurance	Intercept	6		17	5.411 (2.963, 7.858)	4.712	0.001	*F*(1, 15) = 0.29	0	0	8.71	75.51
	Velocity loss	6		17	0.012 (− 0.037, 0.062)	0.535	0.6					
Countermovement jump	Intercept	12		29	0.646 (− 1.498, 2.789)	0.62	0.541	*F*(2, 26) = 6.11	100	13.93	54.84	0
	Velocity loss	12		29	−0.038 (− 0.070, − 0.007)	− 2.503	0.019					
	Study duration	12		29	0.358 (0.087, 0.630)	2.712	0.012					
Sprint	Intercept	9		27	0.093 (− 0.054, 0.239)	1.306	0.204	*F*(2, 24) = 8.62	76.93	33.75	2.93	86.79
	Velocity loss	9		27	0.001 (0.001, 0.002)	3.552	0.002					
	Study duration	9		27	− 0.021 (− 0.040, − 0.002)	− 2.230	0.031					

*CI* confidence interval

^a^Omnibus test

^b^Lower body (reference level)

#### Velocity Against Submaximal (Low and Moderate) Loads

For the final multivariate model investigating the effects of different VL thresholds on velocity against low and moderate loads, seven groups from five studies were identified as influential. Because of the high number of influential groups, these were excluded, and estimates of the model without these influential groups were retained (Fig. 7c). This model revealed VL (*b* = − 0.018 [− 0.029, − 0.006]; *t* = − 3.69; *p* = 0.010) and load (*b* = 1.182 [0.342, 2.022]; *t* = 3.12; *p* = 0.011) as significant moderators (note that low load was a reference outcome). The interaction between the VL and outcome was not significant (*b* = 0.014 [− 0.007, 0.035]; *t* = 1.73; *p* = 0.146). Heterogeneity for the low load outcome was considerably lower (*τ*^2^ = 0.235) compared with the moderate load outcome (*τ*^2^ = 2.034) with the model-estimated correlation between the outcomes being high (*ρ* = 0.844). Imputing a range of different correlations between the low and moderate loads (*ρ* = 0.4–0.8) did not affect the interpretation of the model, confirming the robustness of the estimates.

## Discussion

The present systematic review evaluated the acute effects of different VL thresholds on volume and fatigue during RT and meta-analyzed their chronic effects on training adaptations while considering several factors that might differentially influence the magnitude of these acute and chronic responses. Several interpretations stem from our findings: (1) while the number of repetitions per set generally increases as the VL increases, the variability in repetitions performed is modulated by exercise choice and load and (2) because of these increases in repetitions per set, blood lactate concentration and rating of perceived exertion increase whereas countermovement jump, sprinting, and V1 performance decrease proportionally as VL increases. However, the magnitude of these effects is highly influenced by exercise and load; (3) the specific VL threshold used does not have a profound effect on gains in strength and muscle endurance; however, (4) selecting moderate to high VL thresholds for hypertrophy, and low to moderate thresholds for enhancing countermovement jump, sprint, and velocity against submaximal loads may be a viable strategy to induce superior training adaptations. Therefore, many factors should be considered when prescribing RT using VL thresholds to create more homogeneous stimuli among individuals, thereby optimizing fatigue management and intended training adaptations.

### Effects of Velocity Loss Thresholds on the Number of Repetitions Completed Per Set

Researchers have recommended RT prescription with VL thresholds over traditional methods owing to the strong relationship between the magnitude of VL and the number of repetitions performed with respect to the total number that can be completed before reaching failure [15, 17]. The argument is strengthened by the fact that the number of repetitions performed to failure with a given %1RM has a high inter-individual variability [13]. However, this argument does not discount that the number of repetitions performed before reaching different VL thresholds might also have a high inter-individual variability. Indeed, this contention seems to be empirically supported because data from two recent studies [21, 81] suggest that the number of repetitions performed until reaching 10, 20, and 30% VL in the free-weight back squat exercise is not only highly variable between individuals but is also unstable across sessions. In addition, this inter-individual variability may increase as the magnitude of VL increases [21]. Based on the studies included in the present review, it seems that exercise choice and load can further influence the actual number of repetitions performed and the variability thereof (Fig. 3). Specifically, both the actual number of repetitions and its variability seem to be higher in the back squat compared with the bench press exercise across VL thresholds. Furthermore, both factors tend to have a strong inverse relationship with load, as higher loads allowed for fewer repetitions and produced lower variability in repetitions across VL thresholds. This is a previously overlooked outcome as studies often focus on the ability of VL thresholds to modulate, with acceptable reliability, the percentages of the completed repetitions per set with respect to the maximum number of repetitions possible [15, 17] and kinetic and kinematic outputs [21, 62, 81]. Although these aspects of VL thresholds present an advantage over traditional methods for prescribing RT volume, the effects of the variability of the actual number of repetitions performed before reaching a certain VL threshold have not yet been empirically investigated. It is possible that individuals completing different numbers of repetitions using the same VL threshold might experience different degrees of neuromuscular, metabolic, and perceptual fatigue, potentially influencing resultant training adaptations. In this regard, it is unknown whether the specific VL threshold is a more important variable than the actual number of repetitions performed, as no studies to date have compared different VL thresholds matched for volume. Collectively, based on the studies included in the present review, it seems the use of VL thresholds for RT prescription could result in the considerable variability of the actual number of repetitions per set completed, which can further be confounded by other factors such as the choice of exercise and the load used. Whether this variability could modulate both the acute and chronic effects of VL thresholds presents an interesting avenue for future research.

### Acute Effects of Velocity Loss Thresholds on Neuromuscular, Metabolic, and Perceptual Markers of Fatigue

Fatigue is traditionally defined as a loss of force-generating capacity with the eventual inability to sustain exercise at the required or expected level [82, 83]. Muscle-shortening velocity decreases and relaxation time increases as fatigue ensues [84]. In this regard, velocity against a fixed load (e.g., V1) before and after RT is often used as a marker of neuromuscular fatigue in studies investigating the acute effects of different VL thresholds. Indeed, this marker has a high correlation (*r* > 0.9) with other markers of fatigue such as blood lactate and ammonia accumulation as well as countermovement jump height loss after RT [14–16, 20]. Therefore, it is not surprising that several studies reported an almost linear decrease in post-session V1, and countermovement jump height, as well as an increase in blood lactate accumulation as VL increased [14–16, 21]. However, the dose–response relationship of VL with these markers of fatigue seems to be modulated by the exercise and load used (Fig. 4). For instance, as load decreases while using a given VL threshold, greater reductions in post-session V1 and countermovement jump height are observed [16]. Furthermore, Rodríguez-Rosell et al. [16] observed greater declines in post-session V1 in the bench press compared with the back squat, independent of load and VL. The authors attributed these V1 differences between exercises to the smaller muscles—with more type II fibers and higher fatiguability index—involved in the bench press than the squat exercise [85–87]. Rodríguez-Rosell et al. [16] also reported greater blood lactate accumulation during the back squat compared with the bench press, regardless of the load used and VL experienced. In addition, the rate at which metabolic stress increased, as the VL increased, was considerably lower with greater loads (i.e., 80% RM) during the back squat but not bench press, for which metabolic stress uniformly increased as the VL increased regardless of the load used. Therefore, it seems that VL thresholds induce differential neuromuscular and metabolic responses to RT depending on the exercise used.

One potential explanation for this phenomenon could lie in the actual number of repetitions performed before reaching different VL thresholds. Namely, while the RT protocols employing different exercises used the same VL threshold, it is plausible that performing more work (i.e., more repetitions) until reaching a given VL led to a greater blood lactate accumulation [88, 89]. This is supported by the findings of Weakley et al. [90], which showed greater metabolic responses accompany increases in work completed during RT. Studies included in this review generally show that a higher number of repetitions are completed with the back squat compared with the bench press (Fig. 3). Therefore, when completing more work with the back squat compared with the bench press for a given VL threshold, higher metabolic stress is a logical outcome. Thus, the actual training volume completed in a set with a given VL threshold is an important consideration when prescribing RT. Considering the above, it seems that neuromuscular responses are less sensitive to subtle changes in volume during a set compared with metabolic responses, whereas greater neuromuscular fatigue is induced when using exercises involving smaller muscle groups (greater localized fatigue) with greater percentages of type II muscle fibers (a higher fatiguability index). However, countermovement jump height, also a valid marker of neuromuscular fatigue [91], seems to be extremely sensitive to changes in load (Fig. 4d). As higher loads typically allow for less volume (i.e., repetitions) to be completed in a set, it is plausible that countermovement jump height would also be sensitive to subtle changes in training volume, highlighting that different neuromuscular fatigue assessments might differ in sensitivity. Nevertheless, future research should substantiate these contentions.

Based on the available literature, rating of perceived exertion also seems to increase as VL increases. For instance, Weakley et al. [21] found gradual increases in perceived exertion of the lower limbs and breathlessness after each set with 10, 20, and 30% VL. More specifically, the rate of increase in both perceptual measures seemed to be consistent for the 10% VL threshold, whereas perceived exertion of the lower limbs increased at a greater rate compared with breathlessness across sets with higher VL thresholds (20 and 30%), although the overall magnitude of both perceptual responses was similar. This finding is somewhat supported by Emanuel et al. [92] who reported that the most frequent cause of set termination during sets of back squats to volitional failure was perceived fatigue in the targeted muscles, whereas cardiovascular factors were not as frequent a cause. However, this likely depends on the training background of the individuals. Based on these findings, prescribing larger velocity loss thresholds (e.g., 20 and 30%) for back squats might lead to larger increases in perception of leg muscle exertion than breathlessness across repeated sets. Similar findings were reported by Dos Santos et al. [70] who found that both perceived exertion and discomfort linearly increased as the number of back squat sets increased with a 30% VL threshold. Although it has not been discussed in the literature, the intention of continuously performing repetitions as fast as possible might also impact perceptual responses, especially leg muscle exertion [21] and perceived discomfort [70]. Admittedly, this hypothesis is challenging to investigate as the provision of maximal intent is a prerequisite for reliable velocity outputs.

The time course of fatigue recovery following RT depends on a myriad of factors including training volume and load. Despite the proposed benefits of VL thresholds in the literature [10, 14, 15], only Pareja-Blanco et al. [22] examined the time course of recovery after using different VL thresholds and loads during RT. For this purpose, the researchers examined vertical countermovement jump height, 20-m sprint time, and V1 before RT, and immediately, 6, 24, and 48 h post-back squat training with a combination of 20 and 40% VL and 60 and 80% of 1RM. Interestingly, with 60% 1RM, regardless of the VL used (20 vs 40%), none of the performance tasks fully returned to pre-exercise values at 48 h post-RT. In contrast, the RT protocol using higher loads (80% 1RM) and lower VL (20%) resulted in lower performance impairment immediately after RT, and greater sprint performance at 48 h post-RT compared with baseline. Interestingly, sprint time generally recovered faster compared to countermovement jump height and V1, suggesting their superior sensitivity for detecting RT-induced neuromuscular fatigue, and the fact that recovery may be exercise dependent. Nevertheless, prescribing higher VL (e.g., 40%) and lower relative loads (e.g., 60% 1RM) could result in greater fatigue immediately after RT and a slower rate of recovery than lower VL (e.g., 20%) and higher relative loads (e.g., 80% 1RM). This finding is especially relevant for sports where RT precedes sport-specific training, in which case an appropriate VL may decrease interference with subsequent sports training.

### Methodological Considerations When Implementing Velocity Loss Thresholds and Future Research Directions

Several research groups have suggested that implementing VL thresholds may allow for better fatigue management compared with traditional RT training prescription methods [14, 15]. It also has been suggested VL can serve as a valid indicator of fatigue because of its high correlation with other frequently used neuromuscular and metabolic markers of fatigue [14–17]. While this presents a considerable advancement for RT monitoring and prescription, there are a few methodological factors that could compromise their utility both in research and practice. First, it is not clearly understood when exactly one should terminate a set after reaching a pre-determined VL threshold. In the literature, set termination after either one or two repetitions exceeding a VL threshold is common. The rationale for two repetitions is based on the fact that individuals can in some cases produce a velocity above a certain VL threshold, even after this threshold was exceeded for the first time [24]. On this note, some of the studies included in this review—all of which used VL to prescribe RT—reported considerable variability in the VL achieved at the end of a set (Fig. 4a). The magnitude of this variability reported in several studies [70, 80, 93, 94] ranged from 5 to 13%. At the extreme end of this range, one could theoretically expect an individual to reach 40% VL in a set when only 30 or 35% was intended. These limitations should be considered in practice and future research should investigate ways of reducing this variability. Second, the reference repetition from which the VL is calculated (i.e., the first or the fastest in the set) is an important consideration as it affects the VL achieved and subsequently the number of repetitions performed [24]. As the first repetition is not always the fastest [24, 95, 96], it is important to use the fastest repetition as the reference for VL calculations to ensure more precise RT monitoring and prescription. Third, a reduction in the ability to accelerate the load at the beginning of the concentric phase will likely affect mean velocity more than peak velocity [97, 98]. In this regard, mean velocity should be used rather than peak velocity when implementing VL in training because of its higher sensitivity in detecting the fatigue progression during a set [24]. Fourth, while studies established a close relationship between VL and the percentage of the repetitions completed out of the maximum possible, these percentages may have a high inter-individual variability [24]. In this regard, future research should investigate whether prescribing individualized VL thresholds could circumvent these uncertainties associated with prescribing the same VL for all individuals in a training session. Finally, while the effects of load and exercise selection were thoroughly discussed in the present review, there are other potentially relevant factors such as strength and height of the individual that might affect the utility of VL in practice [99]. Therefore, future research should continue exploring factors that could affect the precision of VL thresholds and subsequent acute and chronic effects of their implementation.

At least some of the limitations already described could be potentially alleviated by establishing the repetitions in reserve (i.e., the specific number of repetitions that remain uncompleted at set termination) velocity relationship. The rationale for establishing the repetitions in reserve velocity relationship is that despite the strong relationship between the percentage of repetitions completed out of the maximum possible with VL, the post-set repetitions in reserve remains unknown when using VL [19]. This is important because the last repetitions of a set contribute more to the alteration of muscle energy balance and the abrupt increase in metabolites such as ammonia [14, 100, 101]. In this regard, two studies attempted to establish the relationship between repetitions in reserve and velocity [19, 102]. Morán-Navarro et al. [19] examined the within-individual variability for the velocity associated with a given number of repetitions in reserve (i.e., 2, 4, 6, and 8) in the Smith machine bench press, shoulder press, bench pull, and back squat. The authors concluded that regardless of the load used, velocity at a given repetition in reserve is very similar and highly reliable for a given exercise. However, within-individual variability was considerably higher for the bench press and shoulder press compared with other exercises, but this variability was lower among more RT-experienced participants. García-Ramos et al. [102] also examined the repetitions in the reserve velocity relationship, and while they found a high correlation for the Smith machine bench press (*r* = 0.88), they also reported large between-individual variability for velocity at a given repetition in reserve (from 1 to 10). Based on these findings, it seems that a repetition in the reserve velocity relationship, like a load velocity profile, should be established for each exercise, and for each individual. Doing so may alleviate many of the shortcomings identified for the VL prescription method. With that said, the literature on this relationship is still scarce with no information available for free-weight exercises, nor on the potential moderating effects of strength, training background, or sex. Considering this, and the conflicting results already reported in the literature, future studies should be conducted to address the potential utility of this RT prescription method.

### Effects of Velocity Loss Thresholds on Muscle Strength, Hypertrophy, and Endurance Training Adaptations

Based on the results of the present meta-regression, the choice of VL during RT does not seem to affect the magnitude of strength gains when controlling for other factors such as choice of exercise, strength levels, and training duration (Table 4; Fig. 5). This is despite the fact that most studies reported considerable differences in training volume that linearly increased as the VL increased. These findings are somewhat in accordance with the meta-analysis by Ralston et al. [103] who found only trivial to small effects (effect size differences: 0.14–0.23) of higher (5+ sets) versus lower (1–4 sets) weekly set volumes on strength gains. However, it must be noted that participants in the majority of studies included in that meta-analysis performed sets to muscle failure. In contrast, different VL groups included in the present review differed not only in training volume, but also proximity to failure in each set. For instance, performing repetitions until 10% VL would result in not only lower training volume, but also more repetitions left in reserve compared with performing repetitions until 30% VL with the same load and exercise. Therefore, the findings of the present review might be used to support both the notion of avoiding training to failure and also not needing to perform high-volume protocols when the aim is to optimize strength gains. Indeed, although the majority of studies included in the present review found no statistically significant differences in strength gains between different VL thresholds, the magnitudes of improvement (as quantified by effect sizes) seem to suggest a slight advantage of low to moderate over high VL thresholds (Fig. 5b). The authors from the several studies [25–27, 60] suggested that an inverted U-shaped relationship might exist between VL experienced in a set and maximal strength gains. For instance, Pareja-Blanco et al. [25, 26] reported that once a moderate VL threshold was exceeded (e.g., 20 or 25% VL), further increases in strength gains were not observed. In addition, higher VL thresholds can cause a decrease in the early rate of force development [26] and a reduction in the expression of fast-twitch muscle fibers [18] following RT. Further, several researchers [25, 26] reported that a 0% VL, meaning performing only one repetition during a set, did not lead to optimal strength gains. Therefore, a minimal VL threshold (e.g., ≥ 5%) is needed to induce optimal strength gains. Considering all the above, low to moderate instead of high VL thresholds should be prescribed when the goal is to optimize neuromuscular adaptations to RT.

In contrast to gains in maximal strength, an increase in VL led to a somewhat linear increase in muscle hypertrophy (Fig. 5c, d). In this regard, a meta-analysis from Schoenfeld et al. [8] found a graded dose–response relationship between training volume and muscle hypertrophy. As training volume concomitantly increases with VL, it is not surprising that moderate, and especially high VL thresholds induced the most muscle hypertrophy. Volume, rather than the VL threshold itself, seems to be the factor driving differences in hypertrophy as illustrated by Andersen and colleagues [29] who observed no significant differences between 15 and 30% VL threshold groups in the only longitudinal VL study examining muscle hypertrophy with equated volume. However, this finding is not universal as some studies found moderate VL (e.g., 20–25%) thresholds to be equally effective as higher (e.g., > 40%) VL thresholds at promoting hypertrophy [25, 26]. These discrepancies were not discussed in the scientific literature but could at least partially be explained by the combination of the following factors: (1) training status of the participants (e.g., slight numerical differences in muscle cross-sectional area at baseline in favour of moderate thresholds) and (2) relatively low training frequency (~ 2×/week), study duration (~ 8 weeks; 16 sessions), and the number of sets (~ 6/week). Thus, moderate VL thresholds should be prescribed when the aim is to optimize hypertrophy without sacrificing neuromuscular adaptations.

Traditionally, performing many repetitions per set has been recommended when the goal is to induce positive muscle endurance adaptations during RT [104, 105]. Similar conclusions were drawn in a more recent meta-analysis [35]. Contrastingly, the results of the present meta-regression suggest that different VL thresholds, and thus varying number of repetitions performed per set, do not seem to modulate gains in muscle endurance during RT (Fig. 7a, b; Table 4). In fact, higher VL thresholds seemed to be slightly less effective at inducing muscle endurance gains (Fig. 7b). This is surprising given the observed differences in training volume that linearly increased as the VL increased. Moreover, one study [79] recently reported that the group who performed bodyweight pull-ups until reaching 25% VL improved muscle endurance in the same exercise (i.e., number of repetitions to failure) slightly more than the 50% VL group despite the differences in training volume. In this regard, studies [25, 26, 43, 44] often hypothesize that the superior gains in maximal strength observed for low to moderate compared to high VL thresholds might be responsible for these findings. This is a plausible explanation as the muscle endurance tests used a fixed load both at baseline and post-intervention, meaning that the group that experienced greater strength gains would perform the strength endurance test with a lower relative load compared with the group that experienced lesser strength gains, thus allowing more repetitions to be performed until failure. Indeed, high correlations (*r* = 0.63–0.71) have been reported between improvements in maximal strength and muscle endurance, which could support this contention [43, 44]. In addition, similar dose–response curves for muscle strength and endurance, but not hypertrophy, were observed in a recent study [106] investigating the effects of training volume on muscle adaptations, which aligns with the results of the present meta-regression. However, a training program with a repetition range that mimics the endurance test generally leads to greater improvements in muscle endurance [107]. In this regard, it is unclear why higher VL thresholds, which generally allow for greater repetitions per set and therefore more closely mimic muscle endurance tests, did not prove to be superior for this outcome. Perhaps the fact that most studies in the present review terminated their muscle endurance tests when the barbell reached ~ 0.50 m·s^−1^ could be responsible for these findings, thus making the test relatively more similar to low to moderate, but not high VL thresholds. Future studies are needed to investigate these possibilities.

### Effects of Velocity Loss Thresholds on Performance of Athletic Tasks and Velocity Against Submaximal Loads

Based on the results of the present meta-regression, there is an inverse relationship between VL and subsequent improvement in countermovement jump and sprint performance. In addition, study duration also seems to modulate the gains in jumping and sprinting performance with longer training interventions leading to greater gains in performance. This finding was observed despite the fact that only two out of ten studies that investigated the effects of VL thresholds on jumping or sprinting performance incorporated sprinting or jumping in their training programs (either directly or through playing sport). Jumping and sprinting improvements were also unrelated to maximal strength gains, which were more similar between VL thresholds compared to athletic task performance. Therefore, some authors concluded the degree of RT transfer to actual physical performance was more dependent on the magnitude of VL attained in the set rather than gains in strength [43, 44]. This contention could be supported by the principle of training specificity [108]. In general, average velocity was higher for low to moderate than high VL thresholds. In this regard, significant correlations were reported between the velocity of the repetitions performed and changes in jumping and sprinting performance [43, 44], supporting the importance of repetition velocity for enhancing high-speed actions such as jumping and sprinting. The inverse could also explain these findings, as the number of repetitions performed at slower velocities was progressively greater as VL increased. Therefore, it could also be argued that the excessive amount of fatigue from high VL interferes with athletic task performance. However, more research is needed to determine the causal factor, as Pérez-Castilla et al. [28] found no significant differences in jumping and sprinting improvement between 10 and 20% VL threshold groups with equated volume, the only study to have controlled for volume. Admittedly, this study lasted only 4 weeks (below the average in the present review), compared with only low to moderate VL thresholds, and included different jumping exercises in their training interventions, all of which could have affected the results.

The findings of the present meta-regression on the effects of different VL thresholds on velocity against submaximal loads might support the importance of actual repetition velocity during RT that is implemented with the intent of improving jumping and sprinting performance. Indeed, improvement in velocity against moderate (< 0.8 m·s^−1^), and especially low loads (> 1 m·s^−1^) progressively increased as the VL decreased (Fig. 7c, d). As lower VL thresholds allow for greater velocities and therefore higher velocity adaptations against low loads, these findings collectively support the training specificity concept in relation to RT transfer to the performance of athletic tasks such as jumping and sprinting. A large degree of variability in velocity against moderate loads was observed, which could probably be explained by the large range of loads that fell into the moderate loads category. Nevertheless, it seems that moderate VL thresholds (Fig. 7d) were slightly more effective compared with low and high VL thresholds at improving velocity against moderate loads, further supporting the principle of training specificity. Collectively, these findings support the idea that training should be informed by changes in an individual’s load-velocity profile, as doing so identifies the specific RT-induced adaptations along the load-velocity curve, thus providing a more comprehensive analysis of RT-induced changes compared to maximal strength changes alone.

### Implications for Training and Research Based on the Findings from Longitudinal Studies

Overall, based on the findings of the present review it can be concluded that (1) while the differences in strength and muscle endurance adaptations between VL thresholds are small, low to moderate VL thresholds may be slightly more effective for inducing these adaptations compared with higher VL thresholds; (2) moderate to high thresholds are likely more effective for muscle hypertrophy compared with lower thresholds; (3) jumping and sprinting performance improve the most following lower VL threshold training; and (4) low to moderate VL thresholds will improve velocity against low loads, whereas moderate thresholds more effectively improve velocity against moderate loads. Considering less time is required when training with low to moderate VL thresholds, potential reductions in early rate of force development [26], percentage of fast-twitch muscle fibers [18], and the likely delayed time course of recovery after RT with high VL thresholds [22], low to moderate VL thresholds should generally be prescribed when the goal is to optimize strength and performance adaptations. These findings are especially relevant for team sports where frequent matches throughout the season and extended competition periods alter the length of the preparatory period and its specific phases, but also for individual sports where athletes often train multiple times a day and need to manage RT fatigue for both event performance and sport-specific training sessions.

It must be noted, however, that it is presently unclear if differential effects of low to moderate and high VL thresholds are indeed due to differences in VL (and therefore repetition velocity and proximity to failure), training volume, or a combination of both. In this regard, only two longitudinal studies equated training volume between different VL thresholds, both of which found no significant differences between groups [28, 29]. Therefore, it may be that differences in training volume are the main drivers of differential adaptations following the use of different VL thresholds. In partial support of this, reductions in type IIx fibers and the rate of force development have been shown to be larger following higher as compared with lower volume training [109]. Nevertheless, future studies should equalize training volume between VL thresholds to isolate their effects from the influence of total volume load to support or refute this contention. Furthermore, no studies investigating the effects of different VL thresholds have manipulated the number of sets. Manipulating the number of sets could be a viable strategy to further increase the effectiveness of low to moderate VL thresholds. Increasing the number of sets while keeping VL low to moderate might yield additional muscle hypertrophy, comparable to higher VL thresholds with fewer sets. Choosing to perform more sets with low to moderate VL thresholds to increase volume, rather than use high VL thresholds, might avoid the aforementioned downsides of high VL thresholds (neuromuscular fatigue, poorer strength, and athletic task performance adaptations) while still producing (or perhaps even amplifying) the observed adaptations associated with low to moderate VL thresholds. Another area in need of study is the periodized use of VL thresholds over time (e.g., low to moderate VL phases following high VL in a linear manner, or used concurrently in an undulating design). Such a multifaceted approach to training does have merit, especially in high-performance settings where multiple training qualities often have to be considered throughout a microcycle or mesocycle. Importantly, in a similar manner to VL thresholds for those who do not have access to velocity-tracking devices, cluster or rest-redistribution set structures may be a viable alternative to maintain high repetition velocity while minimizing neuromuscular fatigue during RT [35, 110, 111]. Indeed, Jukic and Tufano [96] recently reported that rest redistribution allowed almost all repetitions (~ 17.5 out of 18) in a clean pull exercise to be performed above 20% VL regardless of the load used across three sets and therefore suggested that rest redistribution could potentially serve as a free ad-hoc alternative to VL thresholds. However, future research is needed to explore these alternatives with a range of different exercises, loads, and athletic populations. Finally, acute responses to different VL thresholds discussed in the present review should also be considered when implementing them in RT programs as they are also likely to affect the magnitude of RT-induced adaptations.

### Risk of Bias Assessment

Most of the studies included in this review did not provide sufficient information regarding the method of randomization. Further, no studies provided information regarding allocation concealment and no studies pre-registered their protocols on a publicly available registry. As a result, these studies were of unclear risk of order effect, allocation concealment, and selective reporting bias. Therefore, researchers should improve their reporting of this information in future studies. Importantly, some studies also had an unclear risk of attrition bias due to not providing sufficient information as to the number of participants included in the analysis after reporting that some did not complete the entire intervention or all procedures. Future studies should report the predefined criteria for participant exclusion from analysis, and clearly state how many were included. We recommend the use of the CONSORT flow diagram [112]. Almost half of the studies included in this review were at high risk of familiarization bias because the authors did not report or did not familiarize their participants with the testing procedures. This is especially important in the context of velocity-based training where participants need to provide maximal intent during all repetitions to ensure the reliability of velocity outputs. In addition, some studies failed to report details regarding the provision of velocity feedback or encouragement, both of which can affect the findings of a study. Therefore, future research should ensure that familiarization sessions are performed, the procedures are fully reported, and the provision of velocity feedback or encouragement occurs and is documented. Most studies were at a low risk of bias for other factors that could have affected their findings and used valid and reliable methods, equipment, or instruments to evaluate their outcomes of interest.

### Limitations and Considerations

Several aspects of this review should be considered when interpreting the findings. First, the visualizations made from the acute studies and their interpretation are limited by the data reported in the original studies. While attempts were made to perform a meta-analysis of the acute studies, missing data and subsequently authors’ refusal to provide data prevented us from doing so. Second, there were considerably fewer female participants in both the acute and longitudinal studies, which reduces the generalizability of our findings to female participants, and more research on VL thresholds should include female individuals when possible. However, Rissanen et al. [74] recently reported robust and similar increases in strength and power performance in male and female individuals over 8 weeks while performing repetitions until 20% or 40% VL. This suggests that male and female individuals might be responding similarly to different VL thresholds; although, more research is needed to substantiate these claims. Third, while we attempted to consider the moderating effects of study duration, exercise, loads used, and strength levels of the individuals in all meta-analytic models, the number of studies and effect sizes per study meant this could only be performed for some outcomes. For instance, exercises in the vast majority of longitudinal studies were performed in Smith machines. In this regard, the effects of exercise mode (i.e., Smith machine vs free-weight exercises) have not been formally investigated. Therefore, it is presently unknown to what extent the findings of the present review can be translated to scenarios when only free-weight exercises are used, and thus, the findings of this review should be interpreted with this in mind. This also highlights a need for studies that directly compare the acute and chronic effects of different VL thresholds with exercises performed using free weights or using both free weights and Smith machines (while keeping exercises the same) in a cross-over manner. Fourth, some studies did not report all information required for meta-regressions; therefore, we extracted the required information from figures or made estimations (e.g., pre-post assessment correlations) based on other studies. This likely introduced some error and we therefore urge researchers to report standard deviations of differences (and or pre-post assessment correlations) in training intervention studies. In addition, we also urge researchers to respond to data request e-mails and to provide data when there are no legal barriers to doing so. Fifth, a few longitudinal studies estimated 1RM rather than testing 1RM as a measure of maximal strength. Although not ideal, the fact that all these studies were consistent with their procedures before and after the intervention, used load–velocity relationships with high loads (up to 80–95% 1RM), and used Smith machine exercises to predict maximal strength should minimize the impact on their findings. Finally, as there is no consensus regarding the actual velocities attained against low, moderate, and high loads (because these velocities are highly individual), what is considered a “moderate” or “low” load is subjective. Therefore, when interpreting the velocity against submaximal loads outcome in the present review, it should be noted that loads associated with > 1 and < 0.8 m·s^−1^ were classified as low and moderate loads, respectively.

## Conclusions

Monitoring VL during RT may offer additional insights about training response not captured by more traditional methods of prescribing and monitoring RT. However, it is important to note that the acute neuromuscular, metabolic, and perceptual responses to different VL thresholds will likely depend upon the choice of exercise, loads used, number of sets performed, individual athlete characteristics, and more. In addition, factors that can specifically affect the consistency of VL determination such as reference repetition, use of peak or mean velocity, and criteria for set termination (repetitions allowed after the VL is exceeded) should all be considered when implementing VL in practice. Prescribing low to moderate VL thresholds during RT seems to be more time efficient and a generally advantageous strategy compared with higher VL thresholds for optimizing muscle strength and endurance, jumping and sprint performance, as well as velocity against submaximal loads. In contrast, higher VL thresholds may be more effective for promoting muscle hypertrophy. However, prescribing higher VL thresholds during RT can impair rapid force production capability, reduce the expression of fast-twitch muscle fibers, and prolong recovery from RT. In contrast, extremely low VL thresholds can sometimes lead to suboptimal training adaptations. Therefore, low to moderate VL thresholds may be a viable strategy for ensuring optimal performance improvement while preventing the potentially negative effects of fatigue. To conclude, the findings of this review indicate that the specific choice of VL threshold will influence the subsequent RT adaptations, highlighting that VL threshold selection is an important consideration in RT program design.

## Supplementary Information

Below is the link to the electronic supplementary material.

Supplementary file1 (DOCX 13 KB)

Supplementary file2 (DOCX 15 KB)

Supplementary file3 (DOCX 36 KB)

Supplementary file4 (DOCX 54 KB)

## Declarations

### Availability of data and material

The datasets generated and/or analyzed during the current review are available in tables, supplementary files, and at the Open Science Framework (https://osf.io/q4acs/).
