# Supplementary material for: The Acute and Chronic Effects of Implementing Velocity Loss Thresholds During Resistance Training: A Systematic Review, Meta-Analysis, and Critical Evaluation of the Literature
Source: Sports Med. 2022 Sep 30;53(1):177–214. doi: 10.1007/s40279-022-01754-4 (PMC9807551; doi:10.1007/s40279-022-01754-4)
Supplement: Supplementary file 1 — Supplementary file1 (DOCX 13 KB) [file 40279_2022_1754_MOESM1_ESM.docx]

Jukic et al. (2022). The acute and chronic effects of implementing velocity loss thresholds during resistance training: A systematic review, meta-analysis, and critical evaluation of the literature. *Sports Medicine*.

Email corresponding author: ivan.jukic@aut.ac.nz. Sport Performance Research Institute New Zealand (SPRINZ); School of Engineering, Computer and Mathematical Sciences, Auckland University of Technology, Auckland, New Zealand

**Supplementary file I – Systematic search string used in PubMed/MEDLINE**

The following combination of keywords and Boolean operators was used for the systematic search in PubMed/MEDLINE databases: (“velocity loss” OR “velocity threshold” OR “velocity decline” OR “velocity decrement” OR "velocity maintenance") AND (“resistance training” OR “weight training” OR “strength training” OR "squat" OR "bench press" OR "bench-press" OR "deadlift" OR jump* OR plyo* OR “fatigue” OR neuromusc* OR metabol* OR “perceived” OR percept*).
