# Supplementary material for: The Acute and Chronic Effects of Implementing Velocity Loss Thresholds During Resistance Training: A Systematic Review, Meta-Analysis, and Critical Evaluation of the Literature
Source: Sports Med. 2022 Sep 30;53(1):177–214. doi: 10.1007/s40279-022-01754-4 (PMC9807551; doi:10.1007/s40279-022-01754-4)
Supplement: Supplementary file 2 — Supplementary file2 (DOCX 15 KB) [file 40279_2022_1754_MOESM2_ESM.docx]

Jukic et al. (2022). The acute and chronic effects of implementing velocity loss thresholds during resistance training: A systematic review, meta-analysis, and critical evaluation of the literature. *Sports Medicine*.

Email corresponding author: ivan.jukic@aut.ac.nz. Sport Performance Research Institute New Zealand (SPRINZ); School of Engineering, Computer and Mathematical Sciences, Auckland University of Technology, Auckland, New Zealand

**Supplementary file II: Decisions related to calculation of effect sizes of longitudinal studies**

For the maximal strength outcome, Dorrell et al. [75] reported data for back squat, bench press, overhead press, deadlift and back squat, Held et al. [66] for bench press, bench row and deadlift, whereas Rissanen et al. [74] reported data for back squat and bench press only. However, since the majority of other studies included in this review reported lower body exercises, resulting in a slightly disproportionate balance of upper and lower body exercises in the analysis, it was decided that only bench press data from these three studies [75, 66, 74] would be included in the analysis. The fact that other studies investigating upper body exercises almost exclusively used the bench press exercise further influenced our decision. For the hypertrophy outcome, Andersen et al. [29] reported *vastus lateralis* and *rectus femoris* muscle thickness. However, it was decided to only include *vastus lateralis* data in the analysis as other studies more frequently evaluated changes in *vastus lateralis* muscle thickness as their hypertrophy outcome. Importantly, mean changes in muscle thickness were almost identical (1.3 vs 1.4 mm) between the muscles. For the sprint outcome, studies evaluated the effects of different velocity loss thresholds on 10-, 15-, 20-, and 30-meters sprint times with the most frequently examined distance being 20 meters. It was decided that all distances would be included in the analysis, thereby creating a general sprint time outcome. This was done since there were not enough studies to separately analyse different sprinting distances. For the velocity against submaximal loads outcome, studies reported velocity against all common loads (i.e., loads that were identical before and after training interventions) 1) above 1 m/s; 2) below 1 m/s; 3) above 0.8 m/s; and 4) above 0.8 m/s. Since all velocities from the above 0.8 m/s velocity outcome were higher than 1 m/s, and all velocities from the below 1 m/s group were lower than 0.8 m/s, it was decided that two overarching loading groups will be created to simplify the analysis. In this regard, we created and jointly analysed the effects of different velocity loss thresholds on velocity against low (> 1.0 m/s) and moderate (< 0.8 m/s) loads outcomes. However, two studies [28, 29] examined velocity against different percentages of repetition maximum (1RM), ranging from 20 to 80% of 1RM. In these two cases, loads that were < 70% 1RM were classified as low, and loads ≥ 70% as moderate. Finally, during the revision process of this manuscript, one study [74] that also looked at the effects of high vs low velocity loss thresholds on velocity against submaximal loads outcome got published. Unfortunately, the data from that study [74] was not included in the analysis as it would have required further amendments to our predefined criteria regarding overarching loading groups specified above. Namely, the researchers had specific velocity cut-offs of 0.58 and 0.54 m/s for males and females, respectively, in the bench press exercise, and velocity cut-offs of 0.73 and 0.65 m/s for males and females, respectively, in the back squat exercise. While the authors of that study [74] used these cut-offs to distinguish so-called “high” and “low” velocity repetitions (and thus low and high loads, respectively), following our operational definition that was aligned with the rest of the studies included in this review, all the velocity cut-offs in that study (and thus repetitions) would be labelled as “moderate”. This would not be appropriate given the fact that the low velocity repetitions in that study [74] often reached mean values lower than 0.36 m/s. Unfortunately, the loads (i.e., % 1RM) used for the assessment in that study were not specified, so we could not have used our additional criteria specified above. Since the arbitrary decisions regarding what velocity can be achieved against moderate and high loads could have influenced the results, the data from this study were not included in the analysis.
