# Supplementary material for: The Acute and Chronic Effects of Implementing Velocity Loss Thresholds During Resistance Training: A Systematic Review, Meta-Analysis, and Critical Evaluation of the Literature
Source: Sports Med. 2022 Sep 30;53(1):177–214. doi: 10.1007/s40279-022-01754-4 (PMC9807551; doi:10.1007/s40279-022-01754-4)
Supplement: Supplementary file 3 — Supplementary file3 (DOCX 36 KB) [file 40279_2022_1754_MOESM3_ESM.docx]

Jukic et al. (2022). The acute and chronic effects of implementing velocity loss thresholds during resistance training: A systematic review, meta-analysis, and critical evaluation of the literature. *Sports Medicine*. Email corresponding author: ivan.jukic@aut.ac.nz. Sport Performance Research Institute New Zealand (SPRINZ); School of Engineering, Computer and Mathematical Sciences, Auckland University of Technology, Auckland, New Zealand

**Supplementary file III.** Extended summary of the acute studies included in the review.

| **Author (year)** | ***Velocity loss threshold used; Number of sets; load; inter-set rest*** | ***Exercises; load prescription method*** | ***Velocity variable; reference repetition for velocity loss calculation; Number of repetitions performed below the threshold before termination of the set*** | ***Outcomes (methods of assessment)*** |
| --- | --- | --- | --- | --- |
| Banyard et al. (2019) [77] | ▪ 20%; 4.2 ± 0.9 (until reaching a total of 25 reps); 80%1RM; 2 | Free-weight full back-squat; 1RM percentage-based | Mean velocity; velocity of the single repetition performed at 80%1RM in the warm-up; 1 | Mean velocity peak velocity, total time under tension for each repetition and total session, eccentric and concentric time under tension for each repetition and total session (4 linear position transducers); mean force, peak force, mean repetition work, total session work (force plate); mean power and peak power (force × bar velocity); total session load; mean repetition load; average number of repetitions per set |
| García-Sillero et al. (2021) [69] | ▪ 30%; 4; 70%1RM; 3  ▪ 30%; 4; 70%1RM; 3 | Free-weight bench press; 1RM-percentage-based | Mean velocity; fastest repetition; 1 | Mean velocity peak velocity and peak power of the fastest repetition of each set and total session as well as the effort index (linear position transducer); average number of repetitions per set |
| González-García et al. (2020) [63] | ▪ 20%; 2; optimal load (60.9 ± 5.9%1RM); unclear  ▪ 20%; 2; 80%1RM; unclear | Smith machine half back-squat; 1RM percentage-based | Mean velocity; fastest repetition; 1 | Mean velocity (rotatory encoder); CMJ height (force platform); RPE (Borg CR-10 Scale); average number of repetitions per set; training load (set × repetitions × RPE); repetitions per set |
| Muñoz-López et al. (2021) [80] | ▪ 20%; 3; 63.3 ± 2.1% 1RM; 5  ▪ 40%; 3; 63.3 ± 2.1% 1RM; 5 | Smith machine full back-squat; generalised load-velocity relationship-based | Mean propulsive velocity; fastest repetition obtained in the first 3 repetitions; 1 | Mean propulsive velocity (linear encoder); average number of repetitions per set; effort index (fastest velocity achieved in the first exercise set × average velocity loss over the consecutive sets) |
| Nájera-Ferrer et al. (2021) [94] | ▪ 20%; 3; 60%1RM; 2^a^  ▪ 40%; 3; 60%1RM; 2^a^  ▪ 20%; 3; 60%1RM; 2^b^  ▪ 40%; 3; 60%1RM; 2^b^ | Smith machine deep back-squat; 1RM-percentage-based | Mean propulsive velocity; fastest repetition; 1 | Mean propulsive velocity, mean propulsive velocity attained against the absolute load that elicited 1.00 m·s^-1^ (linear velocity transducer); blood lactate concentration (portable lactate analyser); CMJ height (infrared timing system); average number of repetitions per set |
| Nilo Dos Santos et al. (2021) [70] | ▪ 20% (32 ± 7%); 4; 10RM; 2 | Smith machine parallel back-squat; 1RM-percentage-based | Mean propulsive velocity; fastest repetition obtained in the first 3 repetitions; 1 | Mean propulsive velocity (linear velocity transducer); average number of repetitions per set; RPE (OMNI-RES effort scale); rating of discomfort (Borg CR-10 scale). |
| Pareja-Blanco et al. (2019) [22] | ▪ 20%; 3; 60%1RM; 4  ▪ 40%; 3; 60%1RM; 4  ▪ 20%; 3; 80%1RM; 4  ▪ 40%; 3; 80%1RM; 4 | Smith machine full back-squat; generalised load-velocity relationship-based | Mean propulsive velocity; fastest repetition; 1 | Mean propulsive velocity, percent change in velocity loss against the load that elicited a 1 m·s^-1^ (linear velocity transducer); percent change in CMJ height loss (infrared timing system); percent change in running sprint time loss (photocells); average number of repetitions performed during the 3 sets |
| Pearson et al. (2020) [81] | ▪ 10%; 5; ≈ 70%1RM; 3  ▪ 20%; 5; ≈ 70%1RM; 3  ▪ 30%; 5; ≈ 70%1RM; 3 | Free-weight parallel back-squat; generalised load-velocity relationship-based | Mean velocity; velocity reference of 0.70 m·s^-1^; 1 | Mean and peak values of force, power, and velocity for each set (linear position transducer); external load lifted in each set; average number of repetitions per set |
| Rodríguez-Rosell et al. (2018) [16] | ▪ 10%; 3; 50%1RM; 4  ▪ 10%; 3; 60%1RM; 4  ▪ 10%; 3; 70%1RM; 4  ▪ 10%; 3; 80%1RM; 4  ▪ 20%; 3; 50%1RM; 4  ▪ 20%; 3; 60%1RM; 4  ▪ 20%; 3; 70%1RM; 4  ▪ 20%; 3; 80%1RM; 4  ▪ 30%; 3; 50%1RM; 4  ▪ 30%; 3; 60%1RM; 4  ▪ 30%; 3; 70%1RM; 4  ▪ 30%; 3; 80%1RM; 4  ▪ 45%; 3; 50%1RM; 4  ▪ 45%; 3; 60%1RM; 4  ▪ 45%; 3; 70%1RM; 4  ▪ 45%; 3; 80%1RM; 4  ▪ 15%; 3; 50%1RM; 4  ▪ 15%; 3; 60%1RM; 4  ▪ 15%; 3; 70%1RM; 4  ▪ 15%; 3; 80%1RM; 4  ▪ 25%; 3; 50%1RM; 4  ▪ 25%; 3; 60%1RM; 4  ▪ 25%; 3; 70%1RM; 4  ▪ 25%; 3; 80%1RM; 4  ▪ 40%; 3; 50%1RM; 4  ▪ 40%; 3; 60%1RM; 4  ▪ 40%; 3; 70%1RM; 4  ▪ 40%; 3; 80%1RM; 4  ▪ 55%; 3; 50%1RM; 4  ▪ 55%; 3; 60%1RM; 4  ▪ 55%; 3; 70%1RM; 4  ▪ 55%; 3; 80%1RM; 4 | Smith machine full back-squat; generalised load-velocity relationship-based  Smith machine bench press; generalised load-velocity relationship-based | Mean propulsive velocity; fastest repetition; 1 | Mean propulsive velocity, percent change in velocity loss against the load that elicited a 1 m·s^-1^ (linear velocity transducer); blood lactate concentration (portable lactate analyser); average number of repetitions performed during the 3 sets; effort index (fastest velocity of the first exercise set × average velocity loss over all sets) |
| Rodríguez-Rosell et al. (2020) [20] | ▪ 10%; 3; 50%1RM; 4  ▪ 10%; 3; 60%1RM; 4  ▪ 10%; 3; 70%1RM; 4  ▪ 10%; 3; 80%1RM; 4  ▪ 20%; 3; 50%1RM; 4  ▪ 20%; 3; 60%1RM; 4  ▪ 20%; 3; 70%1RM; 4  ▪ 20%; 3; 80%1RM; 4  ▪ 30%; 3; 50%1RM; 4  ▪ 30%; 3; 60%1RM; 4  ▪ 30%; 3; 70%1RM; 4  ▪ 30%; 3; 80%1RM; 4  ▪ 45%; 3; 50%1RM; 4  ▪ 45%; 3; 60%1RM; 4  ▪ 45%; 3; 70%1RM; 4  ▪ 45%; 3; 80%1RM; 4 | Smith machine full back-squat; generalised load-velocity relationship-based | Mean propulsive velocity; fastest repetition; 1 | Mean propulsive velocity, percent change in velocity loss against the load that elicited a 1 m·s^-1^ (linear position transducer), blood lactate concentration (portable lactate analyser); percent change in CMJ height (infrared timing system); effort index (fastest velocity of the first exercise set × average velocity loss over all sets) |
| Sousa-Fortes et al. (2020) [64] | ▪ 20%, 5; 15RM; 3:20 min | Free-weight half back-squat and bench press; 1RM-percentage-based | Mean velocity; unclear; 1 | Mean velocity (linear position transducer); average number of repetitions per set; internal training load (session time x RPE (10-point Borg scale)) |
| Tsoukos et al. (2019) [71] | ▪ 10%; 40%1RM; 1; 0  ▪ 10%; 60%1RM; 1; 0  ▪ 30%; 40%1RM; 1; 0  ▪ 30%; 60%1RM; 1; 0 | Smith machine bench press throw; 1RM percentage-based | Mean velocity; fastest repetition; 1 | Mean propulsive velocity and peak velocity during the protocol, as well as percent change in mean propulsive velocity and peak velocity (linear position transducer); EMG of the pectoralis mayor and triceps brachii (Bipolar Ag/AgCl electrodes); lifted load, number of repetitions, volume load (lifted load × number of repetitions) |
| Tsoukos et al. (2021) [72] | ▪ 10%; 1; 80%1RM; 0  ▪ 30%; 1; 80%1RM; 0 | Smith machine bench press; 1RM-percentage-based | Mean velocity; fastest repetition; 1 | Mean and peak velocity (bench press), as well as the mean propulsive and peak velocity (bench press throw) after the bench press exercise (linear position transducer); EMG after the bench press exercise of the pectoralis major and triceps brachii muscles (Bipolar Ag/AgCl electrodes); number of repetitions; volume load |
| Varela-Olalla et al. (2019) [65] | ▪ 20-27.3%; 1; 40-45%1RM; 0  ▪ 22.1-29.4%; 1; 55-60%1RM; 0  ▪ 20.7-31.1%; 1; 70-75%1RM; 0 | Free-weight bench press; generalised load-velocity relationship-based | Mean velocity; fastest repetition; 2 | Mean velocity (linear transducer); average number of repetitions per set; RPE (OMNI-RES scale). |
| Varela-Olalla et al. (2020) [73] | ▪ 20%; 1; ≈ 85%1RM; 0 | Smith machine half squat; generalised load-velocity relationship-based | Mean propulsive velocity; fastest repetition; 1 | Mean propulsive velocity (linear position transducer); blood lactate concentration (portable lactate analyser); CMJ height (smartphone app); muscle soreness (single-item questionnaire on delayed onset muscle soreness); external load |
| Weakley et al. (2020) [21] | ▪ 10%; 5; ≈ 70%1RM; 3  ▪ 20%; 5; ≈ 70%1RM; 3  ▪ 30%; 5; ≈ 70%1RM; 3 | Free-weight parallel back-squat; generalised load-velocity relationship-based | Mean velocity; velocity reference of 0.70 m·s^-1^;1 | Blood lactate concentration (portable lactate analyser); CMJ, peak concentric velocity, concentric relative peak power output, and impulse at 100 ms of the upward phase (force plate); average number of repetitions per set; Differential-RPE of the lower peripheries and the breathlessness (verbal anchors on the CR100 scale) |
| Weakley et al. (2020) [62] | ▪ 10%; 5; ≈ 70%1RM; 3  ▪ 20%; 5; ≈ 70%1RM; 3  ▪ 30%; 5; ≈ 70%1RM; 3 | Free-weight parallel back-squat; generalised load-velocity relationship-based | Mean velocity; velocity reference of 0.70 m·s^-1^;1 | Mean and peak values of force, power, and velocity for each set (linear position transducer); average number of repetitions per set |

1RM, one-repetition maximum; RPE, rate of perceived effort; CMJ, countermovement jump; EMG, surface electromyographical activation

^a^, endurance training followed by resistance training; ^b^, resistance training followed by endurance training; *, percussion therapy.
