# Supplementary material for: The Acute and Chronic Effects of Implementing Velocity Loss Thresholds During Resistance Training: A Systematic Review, Meta-Analysis, and Critical Evaluation of the Literature
Source: Sports Med. 2022 Sep 30;53(1):177–214. doi: 10.1007/s40279-022-01754-4 (PMC9807551; doi:10.1007/s40279-022-01754-4)
Supplement: Supplementary file 4 — Supplementary file4 (DOCX 54 KB) [file 40279_2022_1754_MOESM4_ESM.docx]

Jukic et al. (2022). The acute and chronic effects of implementing velocity loss thresholds during resistance training: A systematic review, meta-analysis, and critical evaluation of the literature. *Sports Medicine*. Email corresponding author: ivan.jukic@aut.ac.nz. Sport Performance Research Institute New Zealand (SPRINZ); School of Engineering, Computer and Mathematical Sciences, Auckland University of Technology, Auckland, New Zealand

**Supplementary file IV.** Extended summary of the longitudinal studies included in the review

| **Author (reference)** | ***Training protocol (duration in weeks; sessions/w; exercise; loads)*** | ***Velocity loss threshold; number of sets; inter-set rest*** | ***Velocity loss used for all exercises?*** | ***Adherence*** | ***Outcomes (methods of assessment)*** | ***Group-specific outcomes (pre-post intervention changes)*** | ***Comparisons between the groups (outcomes)*** |
| --- | --- | --- | --- | --- | --- | --- | --- |
| Alcazar et al. (2021) [60] | 8; 2; Smith machine full back-squat; from 70%1RM to 85%1RM | ▪ **VL0**; 3; 4  ▪ **VL10**; 3; 4  ▪ **VL20**; 3; 4  ▪ **VL40**; 3; 4 | Yes | 100% | ▪ 1RM, *F_0_*, *v_0_,* P_max_, a, and *a*/*F_0_* (2 force plates synchronised with a linear velocity transducer) | ▪ **VL0:** L-*F_0_* (↑), L-*v_0_* (⟷)*,* L-P_max_ (↑), L-*a* (⟷), H-*F_0_* (↑), H-*v_0_* (↑)*,* H-P_max_ (↑), and H-*a/F_0_* (↓)  ▪ **VL10:** L-*F_0_* (↑), L-*v_0_* (⟷)*,* L-P_max_ (⟷), L-*a* (↓), H-*F_0_* (↑), H-*v_0_* (↑)*,* H-P_max_ (↑), and H-*a/F_0_* (↓)  ▪ **VL20**: L-*F_0_* (↑), L-*v_0_* (⟷)*,* L-P_max_ (↑), L-*a* (⟷), H-*F_0_* (↑), H-*v_0_* (↑)*,* H-P_max_ (↑), and H-*a/F_0_* (⟷)  ▪ **VL40:** L-*F_0_* (↑), L-*v_0_* (⟷)*,* L-P_max_ (↑), L-*a* (⟷), H-*F_0_* (↑), H-*v_0_* (⟷)*,* H-P_max_ (↑), and H-*a/F_0_* (⟷) | **▪ VL0 = VL10 = VL20 = VL40** (L-*F_0_*, L-*v_0_,* L-P_max_, L-*a*, H-*F_0_*, H-*v_0_,* H-P_max_, and H-*a/F_0_*  ▪ **VL0 and VL10 > VL40** (H-*v_0_*)) |
| Andersen et al. (2021) [29] | 9; 2; leg press and leg extension; 85%1RM (leg press) and 75%RM (leg extension) | ▪ **VL15**; 4 (sessions 1-2) and 6 (sessions 3-9) for the leg press and 4 (sessions 1-5) and 6 (sessions 6-9) for leg extension; 2.5  ▪ **VL30**; 2 (sessions 1-2) and 3 (sessions 3-9) for the leg press and 2 (sessions 1-5) and 3 (sessions 6-9) for leg extension; 2.5 | No | 96.1% | ▪ 1RM, mean velocity attained at 30%1RM (MV_30%1RM_), 45%1RM (MV_45%1RM_), 60%1RM (MV_60%1RM_), 75%1RM (MV_75%1RM_), mean power attained at 30%1RM (MP_30%1RM_), 45%1RM (MP_45%1RM_), 60%1RM (MP_60%1RM_), 75%1RM (MP_75%1RM_), as well as *L_0_* and *v_0_* obtained from the load-velocity relationship (linear encoder)  ▪ MVC, RFD for the period between 20% and 80% of MVC (RFD_20-80%MVC_) in addition to 50 ms (RFD_50ms_), 100 ms (RFD_100ms_), and 200 ms (RFD_200ms_) - (force platform)  ▪ Thickness and architecture of VL and RF (B-mode ultrasound) | ▪**VL15:** 1RM (↑), MV_30%1RM_ (⟷), MV_45%1RM_ (⟷), MV_60%1RM_ (⟷), MV_75%1RM_ (↑), MP_30%1RM_ (⟷), MP_45%1RM_ (⟷), MP_60%1RM_ (⟷), MP_75%1RM_ (⟷), *L_0_* (↑), *v_0_* (⟷), MVC (⟷), RFD_20-80%MVC_ (⟷), RFD_50ms_ (⟷), RFD_100ms_ (⟷), RFD_200ms_ (⟷), VL (⟷), RF (⟷), PA (⟷), and FL (⟷)  ▪**VL30:** 1RM (↑), MV_30%1RM_ (⟷), MV_45%1RM_ (⟷), MV_60%1RM_ (⟷), MV_75%1RM_ (↑), MP_30%1RM_ (↑), MP_45%1RM_ (⟷), MP_60%1RM_ (↑), MP_75%1RM_ (⟷), *L_0_* (↑), *v_0_* (⟷), MVC (⟷), RFD_20-80%MVC_ (⟷), RFD_50ms_ (⟷), RFD_100ms_ (⟷), RFD_200ms_ (⟷), VL (⟷), RF (⟷), PA (⟷), and FL (⟷) | **VL15 = VL30** (1RM, MV_30%1RM_, MV_45%1RM_, MV_60%1RM_, MV_75%1RM_, MP_30%1RM_, MP_45%1RM_, MP_60%1RM_, MP_75%1RM_, *L_0_*, *v_0_*, MVC, RFD_20-80%MVC_, RFD_50ms_, RFD_100ms_ and RFD_200ms_; VL, RF, PA, and FL) |
| Dorrell et al. (2020) [75] | 6; 2; back squat, bench press, strict overhead press (only sessions 1, 3, 5, 7, 9, 11, and 12), and deadlift (only sessions 2, 4, 6, 8, 10, 11, and 12); from 70%1RM to 95%1RM | ▪ **VL20** (below the target velocity of each specific zone); 3; unclear | No | 100% | ▪ 1RM (linear position transducer)  ▪ CMJ height (Jump mat) | ▪ **VL20:** back squat 1RM (↑), bench press 1RM (↑) strict overhead press 1RM (↑), deadlift 1RM (↑), CMJ height (↑) | ▪ **VL20:** back squat 1RM (↑), bench press 1RM (↑) strict overhead press 1RM (↑), deadlift 1RM (↑), CMJ height (↑) |
| Fernandez-Ortega et al. (2020) [67] | 12; 3; Smith machine full back-squat and cycle ergometer; 65%1RM (0.70 m·s^-1^) (squat) and 65% of the load applied in the initial assessment (5.3% of body weight) (cycle ergometer) | ▪ **VL20** (squat) RPML20 (cycle ergometer); 4; 3 | No | Unclear | ▪ CMJ and SJ height (infrared timer system)  ▪ T_0-30_ (infrared-light photocell system)  ▪ 1RM  ▪ Maximum power (absolute [P_max_-C] and relative [P_max_-RC]) and velocity (V_max_-C) on the cycloergometer (Wingate test)  ▪ Maximal power (P_max_-S) and velocity (V_max_-S) in the squat (squat test with loads of 30, 40, 45, 60, 70, and 80%1RM) (linear velocity transducer) | ▪ **VL20:** T_0-30_ (↑), 1RM (↑), CMJ height (↑), SJ height (↑), P_max_-C (↑), P_max_-RC (↑), V_max_-C (↑), P_max_-S (↑), V_max_-S (↑) | ▪ **VL20:** T_0-30_ (↑), 1RM (↑), CMJ height (↑), SJ height (↑), P_max_-C (↑), P_max_-RC (↑), V_max_-C (↑), P_max_-S (↑), V_max_-S (↑) |
| Galiano et al. (2020) [78] | 7; 2; Smith machine full back-squat; ~1.14 ± 0.03 m·s^-1^ (~50%1RM) | ▪ **VL5**; 3; 3  ▪ **VL20**; 3; 3 | Yes | 100% | ▪ 1RM, AV, and AV≥1 (linear velocity transducer)  ▪ T_0-20_ (photocells)  ▪ CMJ height (infrared timing system) | ▪ **VL5:** 1RM (↑), AV (↑), AV≥1 (↑), AV<1 (↑), CMJ height (↑), and T20 (↑)  ▪ **VL20:** 1RM (↑), AV (↑), AV≥1 (↑), AV<1 (↑), CMJ height (↑), and T20 (↓) | ▪ **VL5 = VL20** (1RM, AV, AV≥1, AV<1, T20, and CMJ height) |
| Held et al. (2021) [66] | 8; 2; power clean, squat, bench row, deadlift and bench press; 80%1RM | ▪ **VL10**; 4; 2-3 | No | ~94% | ▪ 1RM | ▪ **VL10:** squat 1RM (↑), bench row 1RM (↑), deadlift 1RM (↑), bench press 1RM (↑), and 1RM_total_ (↑) | ▪ **VL10:** squat 1RM (↑), bench row 1RM (↑), deadlift 1RM (↑), bench press 1RM (↑), and 1RM_total_ (↑) |
| Martinez-Canton et al. (2020) [61] | 8; 2; Smith machine full back-squat; from 0.82 m·s^-1^ (~70%1RM) to 0.60 m·s^-1^ (~85%1RM) | ▪ **VL20**; 3; 4  ▪ **VL40** (from VL20 to VL50); 3; 4 | Yes | 100% | ▪ Fatigue test (as many repetitions as possible against 60%1RM load until the velocity felt below 0.50 m·s^-1^), FT-MNR, and FT-AV (linear velocity transducer) |  |  |
| Pareja-Blanco et al. (2017) [18] | 8; 2; Smith machine full back-squat; from 0.82 m·s^-1^ (~70%1RM) to 0.60 m·s^-1^ (~85%1RM) | ▪ **VL20**; 3; 4  ▪ **VL40** (from 20% to 50%); 3; 4 | Yes | 100% | ▪ 1RM, AV, AV>1, and AV<1 (linear velocity transducer)  ▪ T_0-20_ (photocells)  ▪ CMJ height (infrared timing system)  ▪ Muscle volume of QF, RF, VM and VL+VI (1.5-T scanner)  ▪ Muscle CSA (1.5-T scanner)  ▪ Fiber CSA, CSA-I, CSA-IIA, CSA-IIAX, and CSA-IIX (muscle biopsy) | ▪ **VL20:** 1RM (↑), AV (↑), AV>1 (↑), AV<1 (↑), CMJ height (↑), T_0-20_ (⟷), QF (↑), RF (⟷), VM (↑), VL+VI (⟷), CSA (⟷), CSA-I (⟷), CSA-IIA (⟷), CSA-IIAX (⟷), and CSA-IIX (⟷)  ▪ **VL40:** 1RM (↑), AV (↑), AV>1 (=), AV<1 (↑), CMJ height (↑), T_0-20_ (⟷), QF (↑), RF (⟷), VM (↑), VL+VI (↑), CSA (↑), CSA-I (↑), CSA-IIA (⟷), CSA-IIAX (↑), and CSA-IIX (⟷) | ▪ **VL20 = VL40** (1RM, AV<1, T20, QF, RF, and VM)  ▪ **VL20 > VL40** (AV, AV>1, CMJ height, CSA, CSA-I, CSA-IIA, CSA-IIAX, and CSA-IIX)  ▪ **VL40 > VL20** (VL, VI) |
| Pareja-Blanco et al. (2017) [76] | 6; 3; Smith machine full back-squat; from ~1.13 m·s^-1^ (~50%1RM) to ~0.82 m·s^-1^ (~70%1RM) | ▪ **VL15**; 2 (sessions 1, 4, 7, 11, 15, and 18) or 3 (sessions 2, 3, 4, 6, 8, 9, 10, 12, 13, 14, 16, and 17); 4  ▪ **VL30**; 2 (sessions 1, 4, 7, 11, 15, and 18) or 3 (sessions 2, 3, 4, 6, 8, 9, 10, 12, 13, 14, 16, and 17); 4 | No | 85% | ▪ 1RM and AV (linear velocity transducer)  ▪ YIRT  ▪ T_0-30_ (photocells)  ▪ CMJ height (infrared timing system) | ▪ **VL15:** 1RM (↑), AV (⟷), YIRT (↑), T_0-30_ (⟷), and CMJ height (↑)  ▪ **VL30**: 1RM (⟷), AV (⟷), YIRT (↑), T_0-30_ (⟷), and CMJ height (⟷) | ▪ **VL15 = VL30** (1RM, AV, YIRT, and T_0-30_)  ▪ **VL15 > VL30** (CMJ height) |
| Pareja-Blanco et al. (2020) [25] | 8; 2; Smith machine bench press; from 0.65 ± 0.07 m·s^-1^ (70%1RM) to 0.41 ± 0.05 m·s^-1^ (85%1RM) | ▪ **VL0**; 3; 4  ▪ **VL15**; 3; 4  ▪ **VL25**; 3; 4  ▪ **VL50**; 3; 4 | Yes | 100% | ▪ MIF, RFD_max_, slope of the force-time curve obtained over 50 ms (RFD_0-50_), 100 ms (RFD_0-100_), 150 ms (RFD_0-150_), 200 ms (RFD_0-200_), and 400 ms (RFD_0-400_) - (dynamometric platform)  ▪ 1RM, *v_0_* (bar weight < 0.2 kg), AV, AV>0.8, and AV<0.8 (linear velocity transducer)  ▪ Fatigue test (as many repetitions as possible against 70%1RM load until the muscle failure), FT-MNR, and FT-AV  (linear velocity transducer)  ▪ Muscle CSA of PM (B-mode ultrasonography) | ▪ **VL0:** MIF (↑), RFD_max_ (⟷), RFD_0-50_ (↑), RFD_0-100_ (⟷), RFD_0-150_ (⟷), RFD_0-200_ (⟷), RFD_0-400_ (⟷), 1RM (↑), *v_0_* (⟷), AV (↑), AV>0.8 (↑), AV<0.8 (↑), FT-MNR (↑), FT-AV (↑)and, PM (↑)  ▪ **VL15:** MIF (↑), RFD_max_ (⟷), RFD_0-50_ (⟷), RFD_0-100_ (⟷), RFD_0-150_ (⟷), RFD_0-200_ (⟷), RFD_0-400_ (⟷), 1RM (↑), *v_0_* (↑), AV (↑), AV>0.8 (↑), AV<0.8 (↑), FT-MNR (↑), FT-AV (↑), and PM (↑)  ▪ **VL25:** MIF (↑), RFD_max_ (⟷), RFD_0-50_ (⟷), RFD_0-100_ (⟷), RFD_0-150_ (⟷), RFD_0-200_ (⟷), RFD_0-400_ (↑), 1RM (↑), V_0_ (⟷), AV (↑), AV>0.8 (↑), AV<0.8 (↑), FT-MNR (↑), FT-AV (↑), and PM (↑)  ▪ **VL50:** MIF (↑), RFD_max_ (⟷), RFD_0-50_ (⟷), RFD_0-100_ (⟷), RFD_0-150_ (↑), RFD_0-200_ (⟷), RFD_0-400_ (↑), 1RM (↑), V_0_ (⟷), AV (↑), AV>0.8 (↑), AV<0.8 (↑), FT-MNR (↑), FT-AV (↑), and PM (↑) | ▪ **VL0 = VL15 = VL25 = VL50** (MIF, RFD_max_, RFD_0-50_, RFD_0-100_, RFD_0-150_, RFD_0-200_, RFD_0-400_, 1RM, *v_0_*, AV, AV>0.8, AV<0.8, FT-MNR, FT-AV, and PM*)  ***VL50 > VL0** (PM) |
| Pareja-Blanco et al. (2020) [26] | 8; 2; Smith machine full back-squat; from 70%1RM to 85%1RM | ▪ **VL0**; 3; 4  ▪ **VL10**; 3; 4  ▪ **VL20**; 3; 4  ▪ **VL40**; 3; 4 | Yes | 100% | ▪ T_0-10_, T_10-20_, and T_0-20_ (photocells)  - CMJ height (infrared timing system)  ▪ MVIC, RFD_max_, RFD_0-50_, RFD_0-100_, and RFD_0-150_ (dynamometric platform)  ▪ 1RM, AV, AV>1, and AV<1 (linear velocity transducer)  ▪ Fatigue test (as many repetitions as possible against 70%1RM load until the velocity fell below 0.5 m·s^-1^), and FT-MNR (linear velocity transducer)  ▪ Muscle CSA and architecture of VL (B-mode ultrasonography) | ▪ **VL0:** T_0-10_ (⟷), T_10-20_ (⟷), T_0-20_ (⟷), CMJ height (↑), MVIC (↑), RFD_max_ (⟷), RFD_0-50_ (⟷), RFD_0-100_ (⟷), RFD_0-150_ (⟷), 1RM (↑), AV (↑), AV>1 (↑), AV<1 (↑), FT-MNR (↑), CSA (⟷), PA (⟷), and FL (⟷)  ▪ **VL10** = T_0-10_ (⟷), T_10-20_ (⟷), T_0-20_ (↓), CMJ height (↑), MVIC (↑), RFD_max_ (⟷), RFD_0-50_ (⟷), RFD_0-100_ (⟷), RFD_0-150_ (⟷), 1RM (↑), AV (↑), AV>1 (↑), AV<1 (↑), FT-MNR (↑), CSA (⟷), PA (↑), and FL (⟷)  ▪ **VL20** = T_0-10_ (⟷), T_10-20_ (⟷), T_0-20_ (⟷), CMJ height (↑), MVIC (↑), RFD_max_ (⟷), RFD_0-50_ (⟷), RFD_0-100_ (⟷), RFD_0-150_ (⟷), 1RM (↑), AV (↑), AV>1 (↑), AV<1 (↑), FT-MNR (↑), CSA (↑), PA (⟷), and FL (↑)  ▪ **VL30** = T_0-10_ (⟷), T_10-20_ (⟷), T_0-20_ (⟷), CMJ height (↑), MIF (↑), RFD_max_ (⟷), RFD_0-50_ (↑), RFD_0-100_ (⟷), RFD_0-150_ (⟷), 1RM (↑), AV (↑), AV>1 (↑), AV<1 (↑), FT-MNR (↑), CSA (↑), PA (⟷), and FL (↑) | ▪ **VL0 = VL10 = VL20 = VL40** (T_0-10_, T_10-20_, T_0-20_, CMJ, MVIC, RFD_max_*, RFD_0-50_, RFD_0-100_, RFD_0-150_, 1RM, AV, AV>1, AV<1, FT-MNR, CSA, PA, and FL)  ***VL0 > VL10** |
| Pérez-Castilla et al. (2018) [28] | 4; 2; Smith machine countermovement jump; 1.20 m·s^-1^ (~40%1RM) | ▪ **VL10**; the number of sets was extended until completing 36 repetitions; 4  ▪ **VL20**; the number of sets was extended until completing 36 repetitions; 4 | No | 100% | ▪ CMJ height, *F_0_*, *v_0_*, P_max_, and *a* (infrared platform)  ▪ 1RM, MPV attained at 20 (MPV_20_), 40 (MPV_40_), 60 (MPV_60_), and 80 (MPV_80_) kg  ▪ T_0-15_ (photocells) | ▪ **VL10** = *F_0_* (⟷), *v_0_* (⟷), *a* (⟷), P_max_ (⟷), 1RM (⟷), MPV_20_ (↑), MPV_40_ (⟷), MPV_60_ (⟷), MPV_80_ (⟷), CMJ height (↑), and T_0-15_ (⟷)  ▪ **VL20** = *F_0_* (⟷), *v_0_* (⟷), *a* (⟷), P_max_ (⟷), 1RM (⟷), MPV_20_ (⟷), MPV_40_ (⟷), MPV_60_ (⟷), MPV_80_ (⟷), CMJ height (↑), and T_0-15_ (⟷) | ▪ **VL10 = VL20** (*F_0_*, *v_0_*, *a*, P_max_, 1RM, MPV_20_, MPV_40_, MPV_60_, MPV_80_, CMJ height, and T_0-15_) |
| Rissanen et al. (2022) [74] | 8; 2; Smith machine full back-squat and Smith machine bench press; from 65%1RM to 75%1RM | ▪ **VL20 males**; 3 sessions 2-3-6-7-11), 4 (sessions 4-8-12-13) and 5 (sessions 5-9-10-14-15); 3  ▪ **VL40 males**; 3 sessions 2-3-6-7-11), 4 (sessions 4-8-12-13) and 5 (sessions 5-9-10-14-15); 3  ▪ **VL20 females**; 3 sessions 2-3-6-7-11), 4 (sessions 4-8-12-13) and 5 (sessions 5-9-10-14-15); 3  ▪ **VL40 females**; 3 sessions 2-3-6-7-11), 4 (sessions 4-8-12-13) and 5 (sessions 5-9-10-14-15); 3 | No | VL20 males = 98 ± 3%  VL40 males = 97 ± 5%  VL20 females = 95 ± 6%  VL40 females = 95 ± 4% | ▪ back squat and bench press 1RM, AV>0.73 and AV<0.73 (back-squat), AV>0.58 and AV<0.58 (bench press) (linear velocity transducer)  ▪ CMJ height (force plate)  ▪ Muscle CSA of VL (B-mode ultrasonography) | ▪ **VL20** **males** = back-squat 1RM (↑), bench press 1RM (↑), AV>0.73 (↑), AV<0.73 (↑), AV>0.58 (↑), AV<0.58 (↑), CMJ height (↑), and CSA (↑)  ▪ **VL40** **males** = back-squat 1RM (↑), bench press 1RM (↑), AV>0.73 (↑), AV<0.73 (↑), AV>0.58 (↑), AV<0.58 (⟷), CMJ height (⟷), and CSA (↑)  ▪ **VL20** **females** = back-squat 1RM (↑), bench press 1RM (↑), AV>0.73 (↑), AV<0.73 (↑), AV>0.58 (↑), AV<0.58 (⟷), CMJ height (↑), and CSA (↑)  ▪ **VL40** **females** = back-squat 1RM (↑), bench press 1RM (↑), AV>0.73 (↑), AV<0.73 (↑), AV>0.58 (↑), AV<0.58 (↑), CMJ height (↑), and CSA (↑) | ▪ **VL20 males = VL40 males = VL20 females = VL40 females** (back-squat 1RM, bench press 1RM, AV>0.73, AV<0.73, AV>0.58, AV<0.58, CMJ height, and CSA)  ▪ **VL20 males > VL40 males** (AV<0.73)  ▪ **VL40 females > VL20 females** (AV<0.58) |
| Riscart-López et al. (2021) [42] | 8; 2; smith machine full back-squat; from 50%1RM to 85%1RM with increments of 5%1RM every 2 sessions (LP), from 85%1RM to 50%1RM with decreases of 5%1RM every 2 sessions (RP), from 50%1RM to 85%1RM with changes in %1RM every session (UP), and ~67.5%1RM (CP) | ▪ **VL20**; 3; 4 | Yes | 100% | ▪ 1RM, AV, AV>1, and AV<1 (linear velocity transducer)  ▪ T_0-20_ (photocells)  ▪ CMJ height (infrared timing system) | ▪ **LP:** 1RM (↑), AV (↑), AV>1 (↑), AV>1 (↑), T_0-20_ (↓), and CMJ height (↑)  ▪ **RP:** 1RM (↑), AV (↑), AV>1 (↑), AV>1 (↑), T_0-20_ (↓), and CMJ height (↑)  ▪ **UP:** 1RM (↑), AV (↑), AV>1 (↑), AV>1 (↑), T_0-20_ (↓), and CMJ height (↑)  ▪ **CP:** 1RM (↑), AV (↑), AV>1 (↑), AV>1 (↑), T_0-20_ (↓), and CMJ height (↑) | ▪ **LP = RP = UP = CP** (1RM, AV, AV>1, AV>1, T_0-20_, and CMJ height) |
| Rodiles-Guerrero et al. (2020) [27] | 5; 3; weight stack machine bench press; from 0.67 m·s^-1^ (~65%1RM) to 0.39 m·s^-1^ (~85%1RM) | ▪ **VL10**; 4; 3  ▪ **VL30**; 4; 3  ▪ **VL50**; 4; 3 | Yes | Unclear | ▪ 1RM, AV, AV≥0.8, and AV<0.8 (linear velocity transducer) | ▪ **VL10:** 1RM (↑), AV (↑), AV≥0.8 (↑), AV<0.8 (↑)  ▪ **VL30:** 1RM (↑), AV (↑), AV≥0.8 (⟷), AV<0.8 (↑)  ▪ **VL50:** 1RM (↑), AV (↑), AV≥0.8 (⟷), AV<0.8 (↑) | ▪ **VL10 = VL30 = VL50** (1RM, AV, AV≥0.8, and AV<0.8) |
| Rodríguez-Rosell et al. (2020) [43] | 8; 2; Smith machine full back-squat; from ~0.84 m·s^-1^ (~70%1RM) to ~ 0.60 m·s^-1^ (~85%1RM) | ▪ **VL10**; 3; 4  ▪ **VL30**; 3; 4 | Yes | 100% | ▪ 1RM, AV, AV>1, AV<1, MPV attained against 30 kg (MPV_30_), MPV_40_, 50 kg (MPV_50_), MPV_60_, 70 kg (MPV_70_), and MPV_80_ (linear velocity transducer)  ▪ T_0-10_ and T_0-20_ (photocells)  ▪ CMJ height (infrared timing system)  ▪ Fatigue test (as many repetitions as possible against an absolute load move to ~0.84 m·s^-1^ (~70%1RM until the MPV fell below 0.5 m·s^-1^), and FT-MNR (linear velocity transducer) | ▪ **VL10:** 1RM (↑), AV (↑), AV>1 (↑), AV<1 (↑), MPV_30_ (↑), MPV_40_ (↑), MPV_50_ (↑), MPV_60_ (↑), MPV_70_ (↑), MPV_80_ (↑), T_0-10_ (↓), T_0-20_ (↓), CMJ (↑), and FT-MNR (↑)  ▪ **VL30:** 1RM (↑), AV (↑), AV>1 (↑), AV<1 (↑), MPV_30_ (↑), MPV_40_ (↑), MPV_50_ (↑), MPV_60_ (↑), MPV_70_ (↑), MPV_80_ (↑), T_0-10_ (⟷), T_0-20_ (⟷), CMJ height (↑), and FT-MNR (↑) | ▪ **VL10 = VL30** (1RM, AV, AV>1, AV<1; MPV_30_, MPV_40_, MPV_50_, MPV_60_, MPV_70_, MPV_80_, CMJ height, and FT-MNR)  ▪ **VL10 > VL30** (T_0-10_, and T_0-20_) |
| Rodríguez-Rosell et al. (2021) [45] | 8; 2; Smith machine full back-squat; from ~1.16 m·s^-1^ (~50%1RM) to ~0.68 m·s^-1^ (~80%1RM) using LP and UP | ▪ **VL15**; 3; 4 | Yes | 100% | ▪ 1RM, AV, AV>1, and AV<1 (linear velocity transducer)  ▪ CMJ height (infrared timing system)  ▪ Fatigue test (as many repetitions as possible against ~1.16 m·s^-1^ (~60%1RM) load until the MPV fell below 0.5 m·s^-1^), and FT-MNR (linear velocity transducer) | ▪ **LP:** 1RM (↑), AV (↑), AV>1 (↑), AV<1 (↑), CMJ height (↑), and FT-MNR (↑)  ▪ **UP:** 1RM (↑), AV (↑), AV>1 (↑), AV<1 (↑), CMJ height (↑), and FT-MNR (⟷) | ▪ **LP > UP** (1RM, AV, AV>1, AV<1, and FT-MNR)  ▪ **LP = UP** (CMJ height) |
| Rodríguez-Rosell et al. (2021) [44] | 8; 2; Smith machine full back-squat; from ~1.08 m·s^-1^ (~55%1RM) to ~0.84 m·s^-1^ (~70%1RM) | ▪ **VL10**; 3; 4  ▪ **VL30** (from VL20 to VL30); 3; 4  ▪ **VL45** (from VL20 to VL45); 3; 4 | Yes | 100% | ▪ 1RM, AV, AV>1, AV<1, MPV_30_, MPV_40_, MPV_50_, MPV_60_, MPV_60_, and MPV_80_ (linear velocity transducer)  ▪ T_0-10_ and T_0-20_ (photocells timing gates)  ▪ CMJ height (infrared timing system)  ▪ Fatigue test (as many repetitions as possible against ~0.84 m·s^-1^ (~70%1RM) load until the MPV fell below 0.5 m·s^-1^), and FT-MNR (linear velocity transducer) | ▪ **VL10:** 1RM (↑), AV (↑), AV>1 (↑), AV<1 (↑), MPV_30_ (↑), MPV_40_ (↑), MPV_50_ (↑), MPV_60_ (↑), MPV_70_ (↑), MPV_80_ (↑), FT-MNR (↑), CMJ height (↑), T_0-10_ (↓), and T_0-20_ (↓)  ▪ **VL30:** 1RM (↑), AV (↑), AV>1 (↑), AV<1 (↑),MPV_30_ (↑), MPV_40_ (↑), MPV_50_ (↑), MPV_60_ (↑), MPV_70_ (↑), MPV_80_ (↑), FT-MNR (↑), CMJ height (↑), T_0-10_ (⟷), and T_0-20_ (↓)  ▪ **VL45:** 1RM (↑), AV (↑), AV>1 (⟷), AV<1 (↑),MPV_30_ (⟷), MPV_40_ (⟷), MPV_50_ (⟷), MPV_60_ (↑), MPV_70_ (↑), MPV_80_ (↑), FT-MNR (↑), CMJ height (↑), T_0-10_ (⟷), and T_0-20_ (⟷) | ▪ **VL10 > VL30 and VL45** (CMJ height, AV, and AV>1)  ▪ **VL30 = VL45** (CMJ height, AV, and AV>1)  ▪ **VL10 = VL45 = VL30** (T_0-10_, T_0-20_, 1RM, AV<1, MPV_30_, MPV_40_, MPV_50_, MPV_60_, MPV_70_, MPV_80_, and FT-MNR) |
| Sánchez-Moreno et al. (2020) [79] | 8; 2; prone-grip pull-up; body mass | ▪ **VL25**; 2 (sessions 1-3 and 16), 3 (sessions 4-8 and 15) or 4 (sessions 9-14); 3  ▪ **VL50**; 2 (sessions 1-3 and 16), 3 (sessions 4-8 and 15) or 4 (sessions 9-14); 3 | Yes | 95% | ▪ 1RM, AV, and MPV_best_ (linear velocity transducer)  ▪ Fatigue test to failure, FT-MNR and FT-AV (linear velocity transducer) | ▪ **VL25:** 1RM (↑), AV (↑), MPV_best_ (↑), FT-MNR (↑), and FT-AV (↑)  ▪ **VL50:** 1RM (⟷), AV (⟷), MPV_best_ (⟷), FT-MNR (⟷), and FT-AV (⟷) | ▪ **VL25 > VL50** (1RM, AV, and MPV_best_, and FT-AV)  ▪ **VL25 = VL50** (FT-MNR) |

1RM, one-repetition maximum; *a*, slope of the force-velocity relationship; *a*/*F_0_*, curvature of the force-velocity relationship; AV, average velocity attained against all absolute loads common to pre-test and post-test; AV>0.73, average velocity attained against absolute loads that were lifted faster than 0.73 m·s^-1^; AV<0.73, average velocity attained against absolute loads that were lifted slower than 0.73 m·s^-1^; AV>0.58, average velocity attained against absolute loads that were lifted faster than 0.58 m·s^-1^; AV<0.58, average velocity attained against absolute loads that were lifted slower than 0.58 m·s^-1^; AV>0.8, average velocity attained against absolute loads that were lifted faster than 0.8 m·s^-1^; AV<0.8, average velocity attained against absolute loads that were lifted slower than 0.8 m·s^-1^; AV≥1, average velocity attained for absolute loads moved at velocities equal to or faster than 1 m·s^-1^; AV<1, the average velocity attained for absolute loads moved slower than 1 m·s^-1^; CP, constant programming; CSA, cross-sectional area; *F_0_*, maximal force; FL, fascicle length; FT-AV, average velocity attained against the same number of repetitions during the fatigue test; FT-MNR, maximum number of repetitions during the fatigue test; *L_0_*, maximal load; LP, linear programming; MIF, maximal isometric force; MPV, mean propulsive velocity; MPV_best_, the fastest MPV attained without additional weight; MVC, maximal voluntary contraction; PA, pennation angle; P_max_, maximal force; PM, pectoralis major; QF, quadriceps femoris; RF, rectus femoris; RIR, reps in reserve; RP, reverse programming; RFD_max_, maximal rate of force development; T_0-10_, 10-sprint time; T_0-20_, 20-sprint time; T_0-30_, 30-sprint time; T_10-20_, time to cover 10- to 20-m;; T_0-15_, 15-sprint time; UP, undulating programming; ; *v_0_*, maximal velocity; VM, vastus medialis; VL, vastus lateralis; VL##, whereby ## refers to the velocity loss threshold used (e.g., VL20 is 20% velocity loss threshold); VL+VI;, vastus lateralis and vastus intermedius; YIRT, total distance covered in the Yo-Yo Intermittent Recovery Test level 1.

↑ reflects an improvement in performance, ⟷ no significant change, and ↓ a decrease in performance. Andersen et al. [29] did not provide statistical inferences for pre-post changes per group. Therefore, ⟷ reflects a < moderate effect size and ↑ reflects a > moderate effect size.
